# MAP4K4 mediates the SOX6-induced autophagy and reduces the chemosensitivity of cervical cancer

**DOI:** 10.1038/s41419-021-04474-1

**Published:** 2021-12-20

**Authors:** Hongxin Huang, Qin Han, Han Zheng, Mingchen Liu, Shu Shi, Ting Zhang, Xingwen Yang, Zhongqing Li, Qiang Xu, Hongyan Guo, Fengmin Lu, Jie Wang

**Affiliations:** 1 Department of Microbiology & Infectious Disease Center, School of Basic Medical Sciences, Peking University Health Science Center, Beijing, 100191 China; 2 Department of Gynecology and Obstetrics, The Third Hospital of Peking University, Beijing, 100191 China

**Keywords:** Cervical cancer, Macroautophagy

## Abstract

There are nearly 40% of cervical cancer patients showing poor response to neoadjuvant chemotherapy that can be induced by autophagy, however, the underlying mechanism has not yet been fully clarified. We previously found that *Sex-determining region of Y-related high-mobility-group box 6* (*SOX6*), a tumor suppressor gene or oncogene in several cancers, could induce autophagy in cervical cancer. Accordingly, this study aims to investigate the mechanism of SOX6-induced autophagy and its potential significance in the platinum-based chemotherapy of cervical cancer. Firstly, we found that SOX6 could promote autophagy in cervical cancer cells depending on its HMG domain. *Mitogen-activated protein kinase kinase kinase kinase-4* (*MAP4K4*) gene was identified as the direct target gene of SOX6, which was transcriptionally upregulated by binding the HMG domain of SOX6 protein to its double-binding sites within *MAP4K4* gene promoter. MAP4K4 mediated the SOX6-induced autophagy through inhibiting PI3K-Akt-mTOR pathway and activating MAPK/ERK pathway. Further, the sensitivity of cervical cancer cells to cisplatin chemotherapy could be reduced by the SOX6-induced autophagy in vitro and in vivo, while such a phenomenon could be turned over by autophagy-specific inhibitor and MAP4K4 inhibitor, respectively. Moreover, cisplatin itself could promote the expression of endogenous SOX6 and subsequently the MAP4K4-mediated autophagy in cervical cancer cells, which might in turn reduce the sensitivity of these cells to cisplatin treatment. These findings uncovered the underlying mechanism and potential significance of SOX6-induced autophagy, and shed new light on the usage of MAP4K4 inhibitor or autophagy-specific inhibitor for sensitizing cervical cancer cells to the platinum-based chemotherapy.

## Introduction

*Sex-determining region of Y-related high-mobility-group box* (*SOX*) gene family consists of a series of homologous genes, each of which possesses a high-mobility-group (HMG) domain that migrates fast in electrophoresis [1, 2]. SOX proteins can function as transcriptional factors and regulate the transcription of multiple genes through directly binding of its HMG domain to the transcriptional regulation region of the target genes, and participate in several processes of growth and development [3–5]. According to the difference in the amino-acid sequence of HMG domain, SOX proteins can be divided into 8 (A-H) subfamilies [6]. SOX6 belongs to the subfamily D of SOX protein and can recognize the conserved sequence of “(A/T)(A/T)CAA(A/T)G” within the transcriptional regulation region of the target gene with its HMG domain [5]. Apart from its role in regulating growth and development, our and other groups have found that SOX6 can also regulate the proliferation of cancer cells [7–14].

In our previous study, we find that SOX6 can inhibit the proliferation of HeLa cells, a cervical cancer cell line, through p14ARF-HDM2-p53 axis [14]. As we know, cervical cancer is related to human papillomavirus (HPV) infection, and nearly all cervical cancer patients are infected with HPV of different subtypes [15]. Although vaccination is widely used to prevent HPV infection, it will take a long period of time for the vaccine to be effectively protective, and many patients are still diagnosed at the locally advanced stage [16]. In clinical practice, the neoadjuvant chemotherapy followed by radical hysterectomy and lymphadenectomy is a viable and effective treatment option for locally advanced cervical cancer, however, there are still nearly 40% of cervical cancer patients showing poor response to neoadjuvant chemotherapy [17, 18]. It has been reported that the sensitivity of cervical cancer cells to cisplatin chemotherapy can be reduced by autophagy [19], however, the underlying mechanisms of poor response induced by autophagy and how to increase the response of these patients have not yet been fully clarified and solved, respectively. Autophagy is a metabolic process highly conserved in eukaryotic cells, through which cells can maintain homeostasis by degrading the misfolded proteins and injured organelles [20]. Several studies have reported the dual effects of autophagy in tumorigenesis at different stages of tumor development [21, 22]. Except for its role in regulating tumorigenesis, autophagy can also reduce the sensitivity of tumor cells to chemotherapy or even lead to chemotherapy resistance [23, 24].

In this study, we found that SOX6 could induce autophagy in cervical cancer cells, and *mitogen-activated protein kinase kinase kinase kinase-4* (*MAP4K4*) gene was identified as a direct target gene of SOX6. MAP4K4, also known as hepatocyte progenitor kinase-like/germinal center kinase-like kinase (HGK) or Nck-interacting kinase (NIK), is a serine/threonine protein kinase related to S. cerevisiae Sterile 20 (STE20) [25]. Except for its role in embryonic development [26, 27], systemic inflammation, type 2 diabetes and atherosclerosis, MAP4K4 also participates in tumor development and is a negative prognostic indicator in several cancers, including hepatocellular carcinoma, lung adenocarcinoma and prostate cancer [28–34]. However, little is known about its role in autophagy and its value in cancer therapy. In this study, we further found that MAP4K4 could mediate the SOX6-induced autophagy through inhibiting PI3K-Akt-mTOR pathway and activating MAPK/ERK pathway, which could reduce the sensitivity of cervical cancer cells to cisplatin chemotherapy in vitro and in vivo.

## Results

### SOX6 promotes autophagosome formation and autophagic flux depending on its HMG domain

**Fig. 1:**
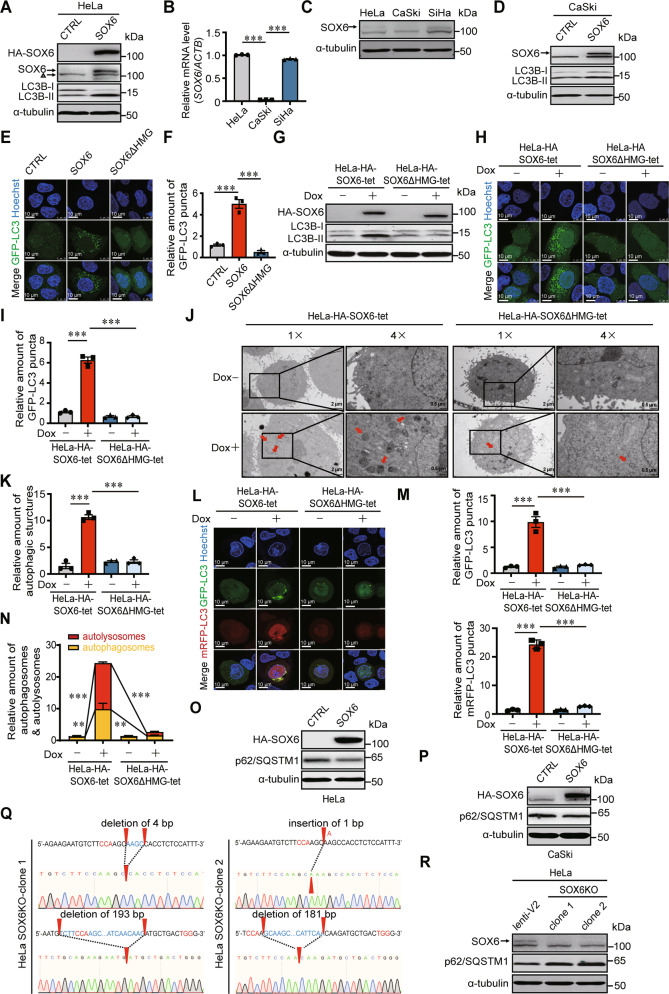
The effects of SOX6 in autophagosome formation and autophagic flux in cervical cancer cells. **A** Western blotting analysis on the level of LC3B protein in HeLa cells transfected with plex-MCS (CTRL) or plex-HA-SOX6 expression plasmid. α-tubulin protein was used as the internal control. △ was used to indicate the non-specific band. **B** The RT-qPCR (SYBR Green) analysis on the levels of SOX6 mRNA in HeLa, CaSki, and SiHa cells. β-Actin (ACTB) mRNA was used as the internal control. **C** Western blotting analysis on the levels of endogenous SOX6 in HeLa, CaSki, and SiHa cells. **D** Western blotting analysis on the level of LC3B protein in CaSki cells transfected with plex-MCS (CTRL) or plex-HA-SOX6 expression plasmid. **E** Representative confocal microscopy images and **F** relative number of GFP-LC3B puncta in HeLa cells co-transfected with GFP-LC3 and plex-MCS (CTRL), plex-HA-SOX6 or plex-HA-SOX6ΔHMG expression plasmids. **G** Western blotting analysis on the level of LC3B protein in HeLa-HA-SOX6-tet and HeLa-HA-SOX6ΔHMG-tet cells treated with or without Dox (4 μg/mL) for 48 h, respectively. **H** Representative confocal microscopy images and **I** relative number of GFP-LC3B puncta in HeLa-HA-SOX6-tet and HeLa-HA-SOX6ΔHMG-tet cells transfected with GFP-LC3 expression plasmid and treated with or without Dox (4 μg/mL) for 48 h, respectively. **J** Transmission electron microscopy analysis and **K** relative amount of autophagic structures (indicated by red arrow) in HeLa-HA-SOX6-tet and HeLa-HA-SOX6ΔHMG-tet cells treated with or without Dox (4 μg/mL) for 48 h. **L** Representative confocal microscopy images, **M** relative amount of GFP-puncta, and **N** relative amount of autophagosomes (GFP-positive, RFP-positive) and autolysosomes (GFP-negative, RFP-positive) in HeLa-HA-SOX6-tet and HeLa-HA-SOX6ΔHMG-tet cells transfected with mRFP-GFP-LC3 adenovirus reporter and treated with or without Dox (4 μg/mL) treatment for 48 h, respectively. **O** Western blotting analysis on the level of p62/SQSTM1 protein in HeLa cells and **P** in CaSki cells transfected with plex-MCS (CTRL) or plex-HA-SOX6 expression plasmid. **Q** The direct sequencing results on the PCR product of *SOX6* gene from two HeLa-SOX6KO clones. The red arrow head indicates the location of the indel (insertion–deletion) mutation in each allele. **R** Western blotting analysis on the levels of p62/SQSTM1 in two HeLa-SOX6KO clones compared with the control HeLa cell (lenti-V2). Data are mean ± SEM of three independent experiments or at least 50 cells scored (**P < 0.01, ***P < 0.001, one-way ANOVA and post hoc Tukey tests).

It has been reported that SOX6 can inhibit the proliferation of HeLa cells in our previous study [14]. In this study, we further found that the level of LC3B-II protein, an autophagy-specific protein, could be increased by overexpressing SOX6 in HeLa cells (Fig. 1A). Since LC3B-II was located on the membrane of autophagosomes and lipidated from the cytosolic LC3B-I, the increased ratio of LC3B-II to LC3B-I (LC3B-II/LC3B-I) induced by SOX6 indicated that SOX6 could promote the formation of autophagosomes (Fig. 1A). Based on the fact that the level of endogenous SOX6 in CaSki cells was far lower than that in HeLa and SiHa cells (Fig. 1B, C), CaSki cells were also chosen to further explore the effect of SOX6 in autophagy of cervical cancer cells. Consistently, SOX6 could also increase the level of LC3B-II/LC3B-I in CaSki cells (Fig. 1D). Further, confocal microscopy analysis showed that the number of GFP-LC3B puncta in HeLa cells overexpressing SOX6 was far more than that in the vector control cells, whereas such a phenomenon was disappeared when its HMG domain was deleted (Fig. 1E, F). Consistently, doxycycline (Dox)-induced SOX6 protein could also increase the level of LC3B-II/LC3B-I and the number of GFP-LC3B puncta in HeLa-HA-SOX6-tet cells, but not for the SOX6 protein with HMG domain deleted (SOX6ΔHMG) in HeLa-HA-SOX6ΔHMG-tet cells (Fig. 1G–I). Moreover, transmission electron microscopy analysis revealed that the Dox-induced SOX6 could increase the number of autophagosome- or autolysosome-like structures in HeLa-HA-SOX6-tet cells, but not in HeLa-HA-SOX6ΔHMG-tet cells (Fig. 1J, K).

Autophagic flux is a dynamic process, including autophagosome formation, autolysosome formation and substrate degradation. To investigate the role of SOX6 in the whole process of autophagic flux, the mRFP-GFP-LC3 adenovirus reporter was used in this study. The result revealed that the number of both yellow (autophagosome) and red puncta (autolysosome) were increased in Dox-treated HeLa-HA-SOX6-tet cells, but not in HeLa-HA-SOX6ΔHMG-tet cells, suggesting that SOX6 could promote the formation of both autophagosome and autolysosome (Fig. 1L–N). As a substrate, p62/SQSTM1 protein is preferentially degraded in autolysosomes [35]. Therefore, to further investigate the role of SOX6 in substrate degradation via autolysosomes, the impact of SOX6 on the level of p62/SQSTM1 protein was analyzed. The result revealed that SOX6 could also significantly reduce the level of p62/SQSTM1 (Fig. 1O, P). Further, *SOX6* gene was knocked out by CRISPR/Cas9 genome editing approach in HeLa cells, and two cell clones with heterozygous insertion-or-deletion (indel) mutations were successfully established (Fig. 1Q). Accompanied by the loss of endogenous SOX6 expression, the autophagic degradation of p62/SQSTM1 was blocked in both HeLa-SOX6KO cell clones (Fig. 1R).

### *MAP4K4* gene is the potential target gene mediating the SOX6-induced autophagy

**Fig. 2:**
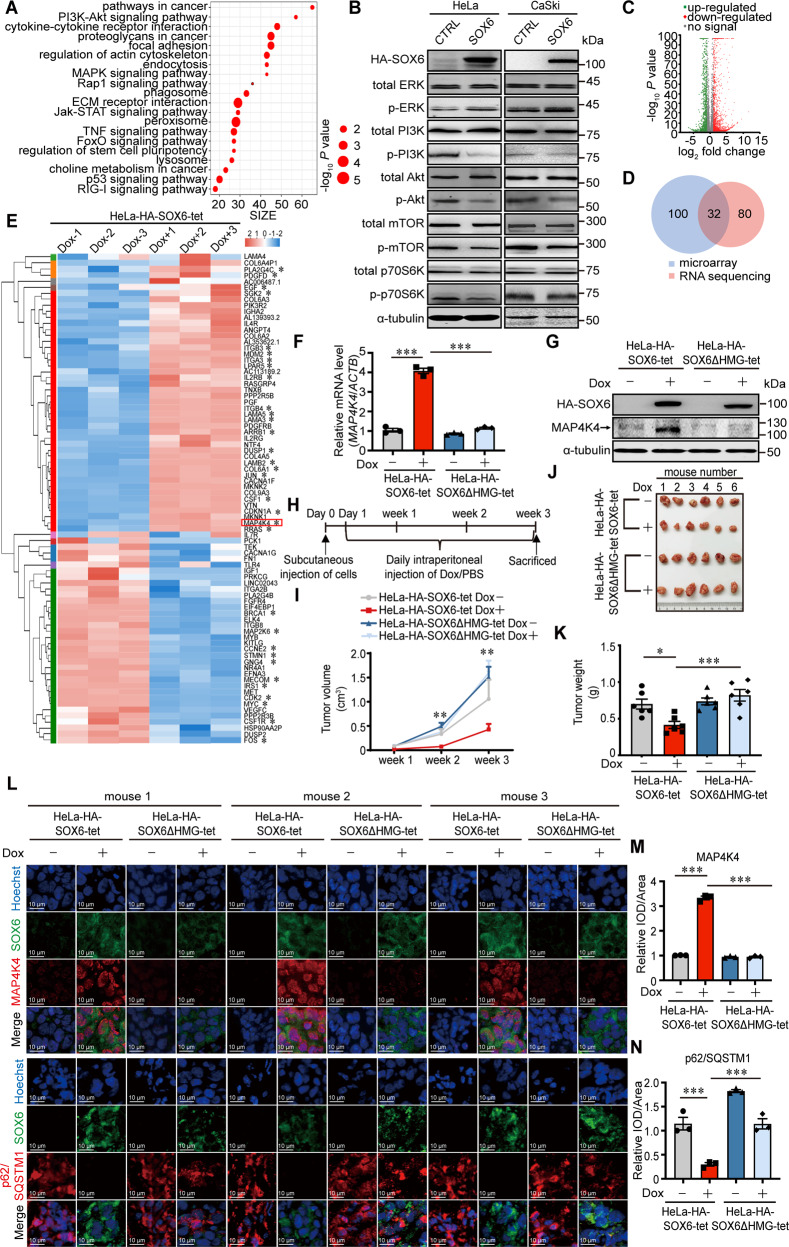
The potential target gene mediating the SOX6-induced autophagy. **A** Scatter plot of top 20 enriched KEGG pathways regulated by SOX6, according to the differentially expressed genes (DEGs) of microarray data. **B** Western blotting analysis on the protein levels of key molecules within MAPK/ERK and PI3K-Akt signaling pathways in HeLa and CaSki cells transfected with plex-MCS (CTRL) or plex-HA-SOX6 expression plasmid. α-tubulin protein was used as the internal control. **C** Overview of the SOX6-regulated DEGs by RNA sequencing. **D** Venn diagram of the DEGs enriched in MAPK and PI3K-Akt signaling pathways between microarray and RNA-sequencing. **E** Heatmap of the 32 DEGs merged between microarray and RNA-sequencing analysis. The color scale beside the heatmap represents the raw *Z*-score ranging from blue (low expression) to red (high expression). **F** The RT-qPCR (SYBR Green) analyses on the levels of MAP4K4 mRNA in HeLa-HA-SOX6-tet and HeLa-HA-SOX6ΔHMG-tet cells treated with or without Dox (4 μg/mL) for 48 h, respectively. **G** Western blotting analyses on the levels of MAP4K4 protein in HeLa-HA-SOX6-tet and HeLa-HA-SOX6ΔHMG-tet cells treated with or without Dox (4 μg/mL) for 48 h, respectively. **H** Experimental design of the steps used to produce xenograft in BALB/c nude mice. **I** Growth curve of tumors formed by subcutaneous injection of HeLa-HA-SOX6-tet or HeLa-HA-SOX6ΔHMG-tet cells into the left flank of nude mice, after which the mice were daily intraperitoneally injected with Dox (20 mg/kg, PBS as solvent control) for 3 weeks. **J** Representative tumor blocks that were collected from the mice sacrificed under anesthesia at 3 weeks post-injection. **K** Average tumor weights (g) of tumor blocks. **L** Immunofluorescence staining of SOX6 (green) and MAP4K4 (red), or SOX6 (green) and p62/SQSTM1 (red) in the frozen section of xenograft tumor tissues. Data are mean ± SEM of at least three fields. **M** Relative expression level of SOX6 (green) and MAP4K4 (red) or **N** SOX6 (green) and p62/SQSTM1 (red) in the frozen section of xenograft tumor tissues. All data are mean ± SEM (*P < 0.05, ***P < 0.001, one-way ANOVA and post hoc Tukey tests). IOD integral optical density.

To investigate the underlying mechanism responsible for SOX6-induced autophagy, genome-wide expression profile of HeLa cells with or without SOX6 overexpression were analyzed. A total of 4488 differentially expressed genes (DEGs) were identified by microarray, and were subsequently analyzed by KEGG pathway enrichment analysis using the Database for Annotation, Visualization and Integrated Discovery (DAVID). The top 20 enriched pathways were listed, and 6 of them were related to autophagy, including PI3K-Akt, MAPK, lysosomal, p53, FoxO, and phagosome pathways (Fig. 2A). According to the number of DEGs, there were most DEGs enriched in PI3K-Akt pathway, followed by MAPK pathway among pathways associated with autophagic machinery (Supplementary Fig. S1). Next, we confirmed that SOX6 could inhibit PI3K-Akt-mTOR pathway, but activate MAPK/ERK pathway in HeLa and CaSki cells (Fig. 2B and Supplementary Fig. S2).

To screen the direct target gene of SOX6 involved in regulating the above two pathways, a total of 2836 DEGs between the HeLa-HA-SOX6-tet cells with and without Dox treatment were further identified by RNA-sequencing analysis (Fig. 2C). Further, 32 overlapping DEGs were screened out by merging the DEGs enriched in PI3K-Akt and MAPK pathways between microarray and RNA-sequencing analysis (Fig. 2D), which were listed in heatmap (Fig. 2E). Among them, *MAP4K4* gene is at the upstream of MAPK signaling pathway and has been reported to regulate both PI3K-Akt and MAPK pathways [36, 37], indicating that *MAP4K4* gene might be the target gene of SOX6 in regulating both pathways. Next, we confirmed that SOX6 could upregulate both mRNA and protein levels of MAP4K4 depending on its HMG domain, which confirmed the results of microarray and RNA-sequencing analyses (Fig. 2F, G, Supplementary Fig. S3A, B). Moreover, the dual-luciferase assay showed that SOX6 could increase the transcriptional activity of the *MAP4K4* gene promoter depending on its HMG domain, suggesting that SOX6 promoted the expression of *MAP4K4* gene at transcriptional level (Supplementary Fig. S4A–C).

Next, the effects of SOX6 in autophagy and the expression of MAP4K4 were further investigated in vivo (Fig. 2H). The result revealed that the Dox-induced SOX6 could significantly reduce the size of tumors in the mice injected with HeLa-HA-SOX6-tet cells at both 2- and 3-weeks post-injection, but not for SOX6ΔHMG in the mice injected with HeLa-HA-SOX6ΔHMG-tet cells (Fig. 2I). Consistently, in the mice injected with HeLa-HA-SOX6-tet cells, the sizes and weights of tumor blocks collected at 3 weeks post-injection were significantly lower in the Dox-treated group than that in PBS-treated group, but not for the mice injected with HeLa-HA-SOX6ΔHMG-tet cells (Fig. 2J, K). Further, SOX6 could also significantly downregulate the level of p62/SQSTM1 protein and upregulate the level of MAP4K4 protein depending on its HMG domain (Fig. 2L–N), which was further confirmed by western blotting analysis (Supplementary Fig. S5).

### *MAP4K4* gene is the direct target gene of SOX6 and mediates the SOX6-induced autophagy

**Fig. 3:**
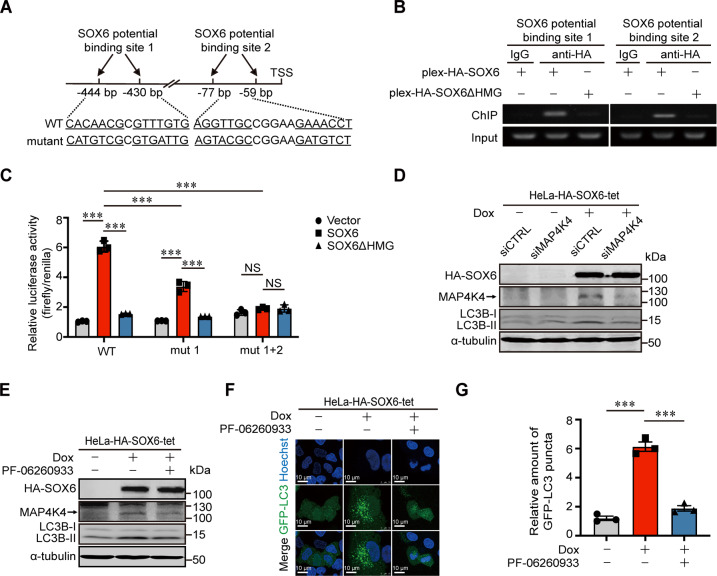
The role of MAP4K4 in the SOX6-induced autophagy. **A** Schematic representation on two potential SOX6 double-binding sites in the promoter region of *MAP4K4* gene. **B** The binding activity of SOX6 to the two potential double-binding sites in the promoter of *MAP4K4* gene was analyzed by ChIP-PCR. **C** Dual-luciferase assay analysis on the effects of SOX6 in the transcriptional activity of wild-type (WT) and mutant (mut 1 and mut 1 + 2) *MAP4K4* gene promoter in HeLa cells. **D** Western blotting analysis on the levels of MAP4K4 and LC3B proteins regulated by SOX6 in HeLa-HA-SOX6-tet cells that the expression of endogenous MAP4K4 was knocked down by transfecting siMAP4K4 or **E** treating with small molecule inhibitor, PF-06260933 (3 μM). α-tubulin protein was used as the internal control. **F** Representative confocal microscopy images and **G** relative number of GFP-LC3 puncta in HeLa-HA-SOX6-tet cells transfected with GFP-LC3 expression plasmid and treated with or without Dox (4 μg/mL) and/or PF-06260933 (3 μM) for 48 h. Data are mean ± SEM and at least 50 cells scored. (***P < 0.001, NS non-significant, one-way ANOVA and post hoc Tukey tests).

Since SOX6-induced autophagy depended on its HMG domain, we further investigated whether *MAP4K4* gene was the direct target gene of SOX6. Firstly, the consensus sequence recognized by HMG domain of SOX6 protein was searched in the promoter region of *MAP4K4* gene. The results revealed that two potential binding sites were identified at 59–77 and 430–444 bp upstream of the transcriptional start site (TSS) of *MAP4K4* gene, respectively (Fig. 3A). Next, chromatin immunoprecipitation (ChIP) combined with PCR assay confirmed that the sequences containing double-binding sites could be bound by the HMG domain of SOX6 protein (Fig. 3B). Further, the dual-luciferase assay showed that SOX6 could significantly enhance the transcriptional activity of *MAP4K4* gene promoter depending on its HMG domain, whereas such an effect was reduced when one binding site was mutated, and was even disappeared when both binding sites were mutated (Fig. 3C). Taken together, the transcriptional activity of *MAP4K4* gene promoter could be enhanced by binding of SOX6 protein to the two binding sites in the promoter of *MAP4K4* gene.

Moreover, the level of LC3B-II/LC3B-I was remarkably reduced when the expression of endogenous MAP4K4 was knocked down by MAP4K4-specific small interfering RNAs (siMAP4K4) (Fig. 3D and Supplementary Fig. S6A) or inhibited by MAP4K4-specific inhibitor (PF-06260933) (Fig. 3E). Similarly, the SOX6-induced autophagosome formation was also inhibited by transfecting siMAP4K4 (Supplementary Fig. S6B) or treating with PF-06260933 (Fig. 3F, G). Above results suggested that MAP4K4 could mediate the SOX6-induced autophagy.

### MAP4K4 mediates the SOX6-induced activation of MAPK/ERK pathway and inhibition of PI3K-Akt-mTOR pathway

**Fig. 4:**
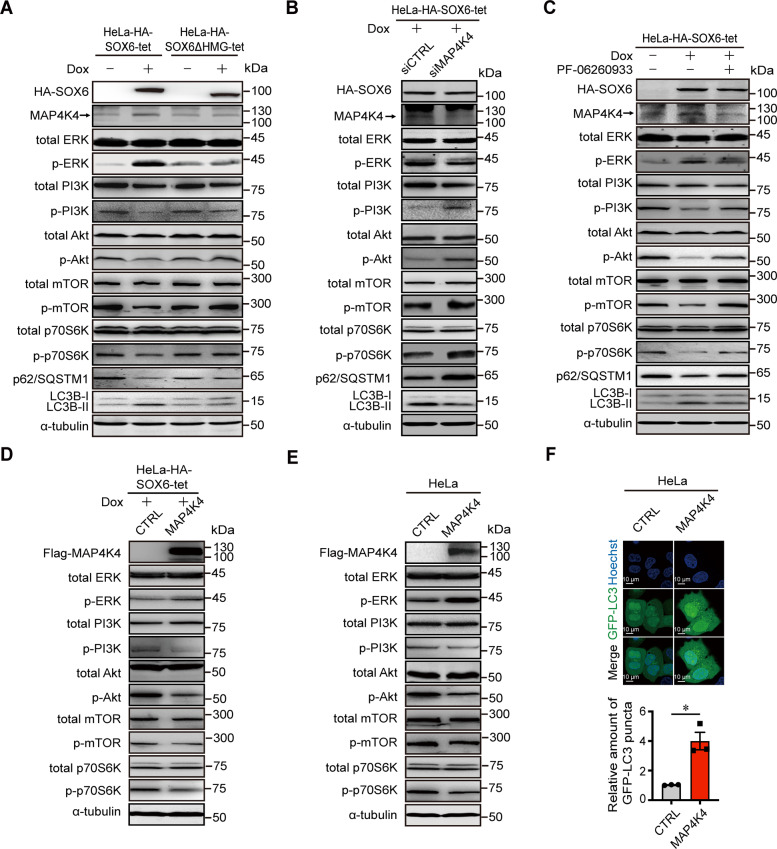
The role of MAP4K4 in the SOX6-induced activation of MAPK/ERK pathway and inhibition of PI3K-Akt-mTOR pathway. **A** Western blotting analysis on the protein levels of key molecules within MAPK/ERK and PI3K-Akt-mTOR signaling pathways in HeLa-HA-SOX6-tet and HeLa-HA-SOX6ΔHMG-tet cells. **B** Western blotting analysis on the protein levels of key molecules within MAPK/ERK and PI3K-Akt-mTOR signaling pathways in HeLa-HA-SOX6-tet cells that the endogenous MAP4K4 was knocked down by transfecting siMAP4K4 or **C** treating with small molecule inhibitor, PF-06260933 (3 μM). **D** Western blotting analysis on the protein levels of key molecules within MAPK/ERK and PI3K-Akt-mTOR signaling pathways in HeLa-HA-SOX6-tet cells transfected with pCDH-MCS (CTRL) or pCDH-Flag-MAP4K4 expression plasmids under Dox (4 μg/mL) treatment for 48 h. **E** Western blotting analysis on the protein levels of key molecules within MAPK/ERK and PI3K-Akt-mTOR signaling pathways in HeLa cells transfected with pCDH-MCS (CTRL) or pCDH-Flag-MAP4K4 expression plasmids, respectively. α-tubulin protein was used as the internal control. **F** Representative confocal microscopy images of HeLa cells co-transfected with GFP-LC3 and pCDH-MCS (CTRL) or pCDH-Flag-MAP4K4 expression plasmids. Data are mean ± SEM and at least 50 cells scored. (*P < 0.05, Student’s *t*-test, two tails).

Since SOX6 could regulate PI3K-Akt-mTOR and MAPK/ERK pathways, and both pathways have been reported to be associated with autophagy [38, 39], we further investigated whether these two pathways were involved in the SOX6-induced autophagy and were regulated by MAP4K4. The result revealed that the Dox-induced SOX6 could activate MAPK/ERK pathway and inhibit PI3K-Akt-mTOR pathway in HeLa-HA-SOX6-tet cells, but not for SOX6ΔHMG in HeLa-HA-SOX6ΔHMG-tet cells (Fig. 4A and Supplementary Fig. S7A–E). Further, the SOX6-induced activation of MAPK/ERK pathway and inhibition of PI3K-Akt-mTOR pathway, as well as autophagy were turned over when the expression of endogenous MAP4K4 was knocked down by siMAP4K4 (Fig. 4B and Supplementary Fig. S7F–J) or inhibited by PF-06260933 (Fig. 4C).

Meanwhile, the SOX6-mediated activation of MAPK/ERK pathway and inhibition of PI3K-Akt-mTOR pathway could be enhanced by ectopic MAP4K4 (Fig. 4D). Moreover, MAP4K4 could also directly activate MAPK/ERK pathway and inhibit PI3K-Akt-mTOR pathway (Fig. 4E), as well as promote the formation of autophagosomes in HeLa cells (Fig. 4F). It suggested that MAP4K4 could mediate the SOX6-induced autophagy through activating MAPK/ERK pathway and inhibiting PI3K-Akt-mTOR pathway.

### SOX6-induced autophagy reduces the sensitivity of cervical cancer cells to cisplatin treatment

**Fig. 5:**
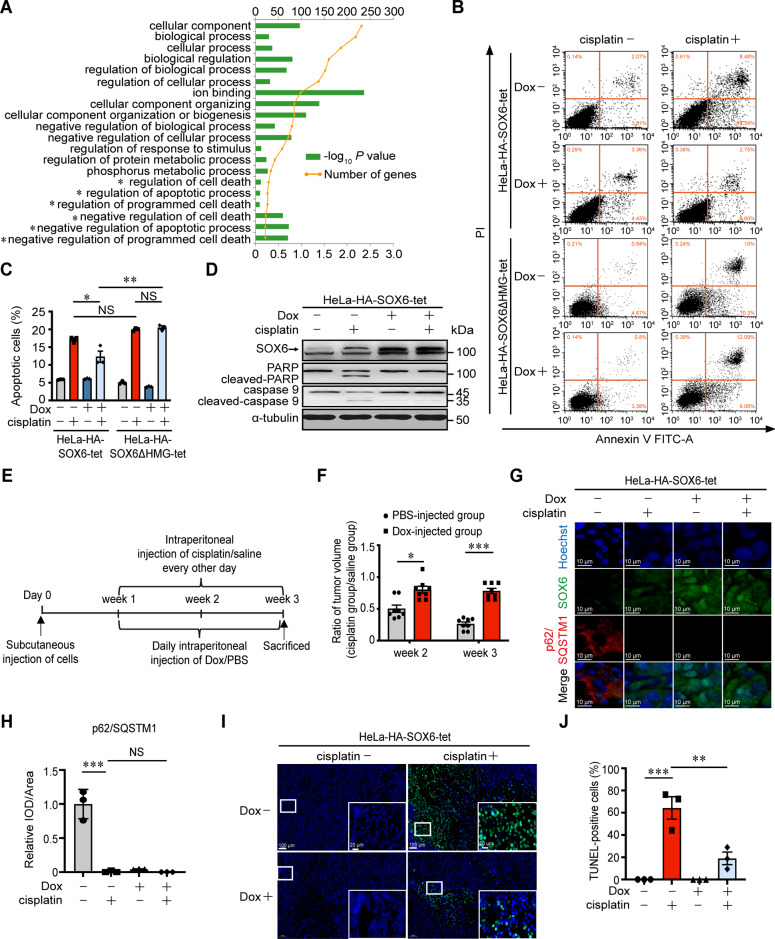
The effect of SOX6-induced autophagy in the sensitivity of cervical cancer cells to cisplatin treatment in vitro and in vivo. **A** Top 20 Gene Ontology (GO)-enriched terms of *MAP4K4* gene. *indicated the terms related to apoptosis and cell death. **B** Flow cytometry analysis on the apoptosis of HeLa-HA-SOX6-tet and HeLa-HA-SOX6ΔHMG-tet cells with or without cisplatin (20 μM) treatment for 48 h. **C** The percentage of apoptotic cells in flow cytometry analysis. **D** Western blotting analysis on the protein levels of PARP and caspase 9 in HeLa-HA-SOX6-tet cells with or without cisplatin (20 μM) treatment. α-tubulin protein was used as the internal control. **E** Experimental design of the steps used to produce xenograft in BALB/c nude mice. **F** Subcutaneously injection of the HeLa-HA-SOX6-tet cells into the left flank of nude mice, which were subsequently divided into two groups at 1-week post-injection and were intraperitoneally injected with Dox (20 mg/kg, PBS as solvent control) every day and cisplatin (3 mg/kg, saline as solvent control) every other day for the next 2 weeks. The relative sizes of the tumors were represented by the ratio of the tumor size in cisplatin group to those in saline group. **G** Immunofluorescence staining of SOX6 (green) and p62/SQSTM1 protein (red) in the frozen section of xenograft tumor tissues. **H** Relative expression level of p62/SQSTM1 indicated by fluorescent density. IOD integral optical density. **I** Immunofluorescence staining of TUNEL (green) in the frozen section of xenograft tumor tissues. **J** The percentage of TUNEL-positive cells in the frozen section of xenograft tumor tissues. Data are mean ± SEM of three independent experiments or at least three fields scored. (*P < 0.05, **P < 0.01, ***P < 0.001, NS non-significant, Student’s *t*-test, two tails for **F**, one-way ANOVA and post hoc Tukey tests for **C**, **H**, **J**).

It has been reported that the cisplatin-mediated anticancer effects are associated with several intertwined mechanisms including DNA damage and subsequent apoptosis, but autophagy could lead to the occurrence of chemoresistance through decreasing the cisplatin-induced apoptosis [40–42]. In this study, we found that some of top 20 Gene Ontology (GO)-enriched terms of *MAP4K4* gene were related to the regulations of apoptosis and cell death (Fig. 5A). Therefore, to further investigate whether the SOX6-induced autophagy affected the therapeutic effect of cisplatin in cervical cancer cells, the effect of SOX6 in the cisplatin-induced apoptosis was firstly analyzed by flow cytometry analysis. The result revealed that SOX6, other than SOX6ΔHMG, could significantly reduce the percentage of cisplatin-induced apoptotic cells (Fig. 5B, C). Further, western blotting analysis confirmed that SOX6 could significantly reduce the levels of apoptosis-related proteins in HeLa-HA-SOX6-tet cells (Fig. 5D), but not for SOX6ΔHMG in HeLa-HA-SOX6ΔHMG-tet cells (Supplementary Fig. S8A).

Next, we further investigated the impact of SOX6-induced autophagy on the chemosensitivity of cisplatin treatment in xenograft (Fig. 5E). The results revealed that the sizes of tumors were significantly reduced at both 2- and 3-weeks post-injection under cisplatin treatment in PBS group, whereas the reduced tumor sizes under cisplatin treatment were significantly lower in Dox-treated group than that in PBS-treated group, indicating that SOX6 could significantly reduce the chemosensitivity of xenograft to cisplatin (Fig. 5F and Supplementary S8B–G). To further investigate the role of SOX6-induced autophagy, the frozen sections of xenograft samples were used for immunofluorescence staining. The results revealed that SOX6 could also induce autophagy in vivo, which was indicated by the decreased level of p62/SQSTM1 (Fig. 5G, H). Meanwhile, the SOX6-induced autophagy could significantly reduce the percentage of apoptotic (TUNEL-positive) cells induced by cisplatin treatment (Fig. 5I, J). These results suggested that the SOX6-induced autophagy could also reduce the chemosensitivity of xenograft to cisplatin in vivo.

### The sensitivity of cervical cancer cells to cisplatin treatment can be increased by inhibiting autophagy and MAP4K4 expression

**Fig. 6:**
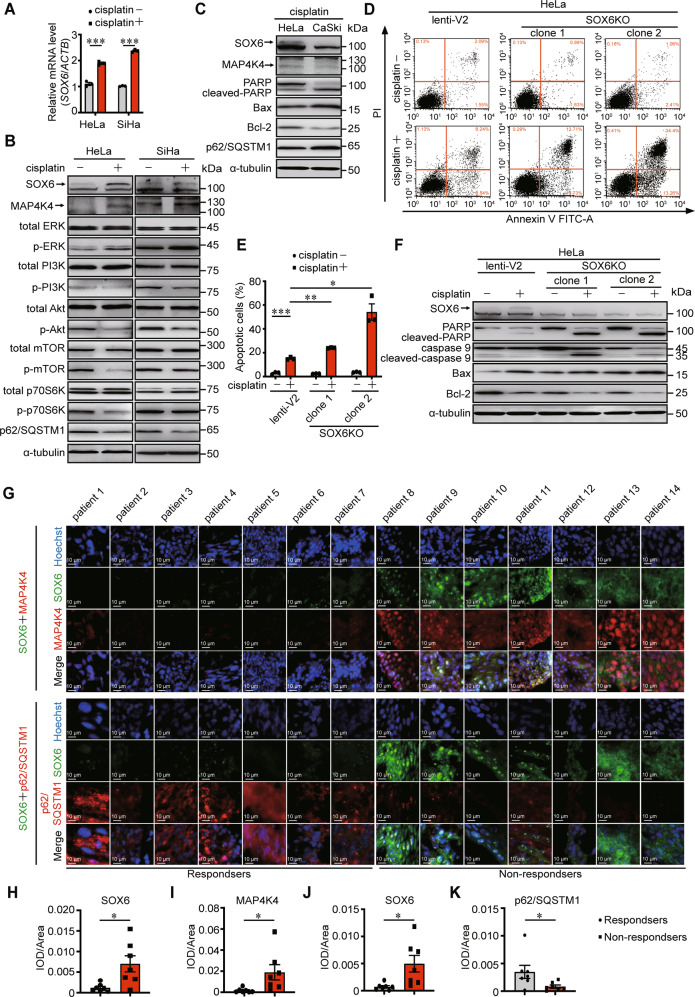
The effect of endogenous SOX6 and MAP4K4 in the sensitivity to cisplatin treatment. **A** The RT-qPCR (SYBR Green) analysis on the levels of SOX6 mRNA in HeLa and SiHa cells that were treated with or without cisplatin (20 μM). β-Actin (ACTB) mRNA was used as the internal control. **B** Western blotting analysis on the protein levels of key molecules within MAPK/ERK and PI3K-Akt-mTOR signaling pathway in HeLa and SiHa cells that were treated with or without cisplatin (20 μM). α-tubulin protein was used as the internal control. **C** Western blotting analysis on the protein levels of SOX6, MAP4K4, PARP, Bax, Bcl-2, and p62/SQSTM1 in HeLa and CaSki cells treated with or without cisplatin (20 μM) treatment. **D** Flow cytometry analysis of apoptosis in two clones of HeLa-SOX6KO cells and the control HeLa cells (lenti-V2) with or without cisplatin (20 μM) treatment. **E** The percentage of apoptotic cells in flow cytometry analysis. **F** Western blotting analysis on the protein levels of PARP, caspase 9, Bax, and Bcl-2 in two clones of HeLa-SOX6KO cells and the control HeLa cells (lenti-V2) cells with or without cisplatin (20 μM) treatment. **G** Representative immunofluorescent staining of SOX6 (green) and MAP4K4 (red) or p62/SQSTM1 (red) in samples collected from 14 cervical cancer patients with routine cisplatin treatment. **H** Relative expression level of SOX6 indicated by fluorescent density. **I** Relative expression level of MAP4K4 indicated by fluorescent density. **J** Relative expression level of SOX6 indicated by fluorescent density. **K** Relative expression level of p62/SQSTM1 indicated by fluorescent density. Data are the mean ± SEM of three independent experiments or at least three fields scored (*P < 0.05, **P < 0.01, ***P < 0.001, Student’s *t*-test, two tails for **A**, **E**, one-way ANOVA and post hoc Tukey tests for **H**–**K**). IOD, integral optical density.

To further investigate the role of SOX6-induced autophagy in the response to cisplatin treatment, we explored the impact of cisplatin treatment on the expression of endogenous SOX6. The results revealed that the level of endogenous SOX6 was significantly increased under cisplatin treatment, and accompanied with the increased level of MAP4K4, activation of MAPK/ERK, inhibition PI3K-Akt-mTOR pathway and induction of autophagy in both HeLa and SiHa cells (Fig. 6A, B and Supplementary Fig. S9). Accompanied with the lower levels of endogenous SOX6 protein and the higher level of p62/SQSTM1 protein in CaSki cells compared to HeLa cells under cisplatin treatment, the sensitivity to cisplatin was higher in CaSki cells than that in HeLa cells, indicating the possibility that the cisplatin-induced higher level of endogenous SOX6 might contribute to the lower sensitivity to cisplatin in HeLa cells (Fig. 6C). This possibility was further confirmed by the results that the sensitivity to cisplatin was significantly increased when the endogenous SOX6 was knocked out in HeLa cells (Fig. 6D–F).

Next, to further verify the clinical significance of SOX6 and MAP4K4 protein levels in cervical cancer patients with routine cisplatin treatment, we collected the cervical cancer tissues from 14 cervical cancer patients after receiving routine cisplatin treatment, half of whom were sensitive to chemotherapy according to the new guidelines to evaluate the response to treatment in solid tumors [43]. The corresponding clinical characteristics were shown in Supplementary Fig. S10. The results revealed that the levels of SOX6 protein and its downstream MAP4K4 protein in the frozen cervical cancer tissues of patients who were sensitive to cisplatin were lower than those in patients who were not sensitive to cisplatin (Fig. 6G–I). Meanwhile, the level of p62/SQSTM1 protein in cervical cancer patients who were sensitive to cisplatin was higher than that in patients who were not sensitive to cisplatin, which further confirmed that the higher level of SOX6-induced autophagy mediated by MAP4K4 might reduce the sensitivity of cervical cancer cells to cisplatin chemotherapy in cervical cancer patients (Fig. 6G, J, K).

**Fig. 7:**
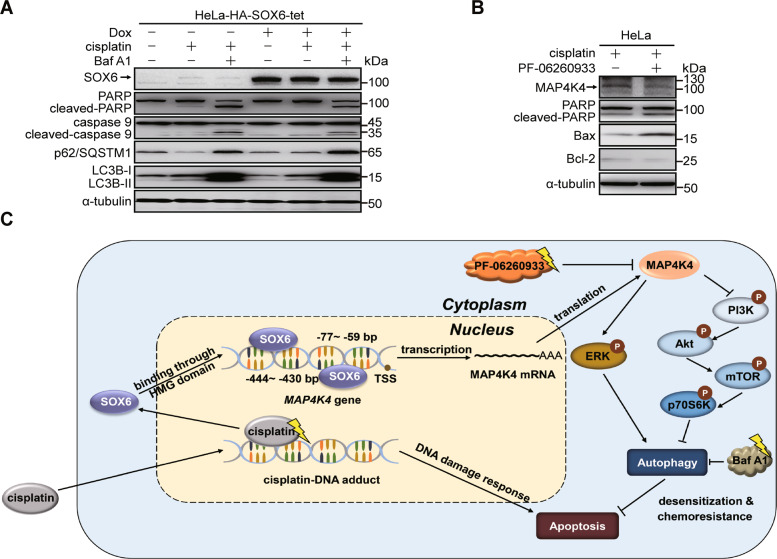
The effects of inhibiting autophagy and MAP4K4 expression in the sensitivity of cervical cancer cells to cisplatin treatment. **A** Western blotting analysis on the protein levels of SOX6, PARP, caspase 9, p62/SQSTM1 and LC3B in HeLa-HA-SOX6-tet cells treated with or without Dox (4 μg/mL), cisplatin (20 μM) for 48 h and/or Baf A1 (10 nM) for the last 6 h. α-tubulin protein was used as the internal control. **B** Western blotting analysis on the protein levels of MAP4K4, PARP, Bax, and Bcl-2 in HeLa cells under cisplatin (20 μM) treatment and treated with or without PF-06260933 (3 μM). **C** The graphical abstract on the mechanism and clinical significance of SOX6-induced autophagy in cervical cancer cells.

Based on the phenomenon that the SOX6-induced autophagy mediated by MAP4K4 could reduce the sensitivity of cervical cancer cells to cisplatin treatment, we further investigated whether the sensitivity to cisplatin could be increased by inhibiting autophagy or MAP4K4 expression. Firstly, Baf A1 was used to inhibit the SOX6-induced autophagy. The result revealed that the SOX6-reduced sensitivity of HeLa-HA-SOX6-tet cells to cisplatin was turned over under Baf A1 treatment (Fig. 7A).

Further, the sensitivity of HeLa cells to cisplatin could also be increased by inhibiting the expression of endogenous MAP4K4 under PF-06260933 treatment (Fig. 7B). Taken together, these results suggested that the sensitivity of cervical cancer cells to cisplatin could be increased by inhibiting SOX6-induced autophagy through both autophagy and MAP4K4 inhibitors.

## Discussion

Cancer chemotherapy is curative in treating a wide range of tumors by inhibiting proliferation and inducing apoptosis of tumor cells [44, 45]. As one of the platinum-based chemotherapeutic agents, cisplatin is widely used in the treatment of advanced cervical cancer, and it can exert anti-tumor function by inducing DNA damage, cell cycle arrest and apoptosis [40, 46]. However, high incidence of mortality is still observed due to the low sensitivity to chemotherapeutic agents or even drug resistance [47]. Therefore, it seems necessary to enhance the sensitivity of tumor cells to chemotherapeutic agents and search for the markers that can possibly predict the efficacy of chemotherapy. Autophagy is a highly conserved process in eukaryotic cells and is regarded as a “double-edged sword” exerting protective or detrimental effects in tumorigenesis [48, 49].

In this study, we firstly found that SOX6-induced autophagy of cervical cancer cells depending on its HMG domain, and *MAP4K4* gene was identified as the potential target gene of SOX6 through microarray and RNA-sequencing analyses. Next, two potential binding sites of SOX6 were identified at 430–444 and 59–77 bp upstream of the TSS of *MAP4K4* gene, and SOX6 could enhance the transcriptional activity of *MAP4K4* gene promoter by binding two binding sites with its HMG domain, suggesting that *MAP4K4* gene was the target gene of SOX6. Further, we found that MAP4K4 not only could mediate the SOX6-induced autophagy, but also directly induce autophagy though activating MAPK/ERK pathway and inhibiting PI3K-Akt-mTOR pathway. Furthermore, SOX6 could significantly inhibit the cisplatin-induced apoptosis of cervical cancer cells dependent on its HMG domain in vitro and in vivo. In turn, the expression of endogenous SOX6 could be upregulated under cisplatin treatment. Based on the higher sensitivity of CaSki cells to cisplatin compared to HeLa cells, there was the possibility that the cisplatin-induced higher level of endogenous SOX6 might contribute to the lower sensitivity to cisplatin in HeLa cells. In line with this possibility, the sensitivity of HeLa cells to cisplatin was increased when the endogenous SOX6 was knocked out, thus the level of SOX6 might be able to predict the efficacy of cisplatin chemotherapy to cervical cancer cells. Moreover, the role of SOX6-induced autophagy was explored in cervical cancer patients who underwent routine neoadjuvant chemotherapy. For the patients who were sensitive to chemotherapy, the levels of SOX6 and its downstream MAP4K4 proteins were lower, but the levels of p62/SQSTM1 were higher than those in patients who were not sensitive to cisplatin, which further confirmed that the high level of SOX6-induced autophagy mediated by MAP4K4 might reduce the sensitivity of cisplatin chemotherapy.

Finally, we further investigated how to increase the sensitivity of cervical cancer cells to cisplatin based on the existing compounds targeting the pathways of SOX6-induced autophagy. Firstly, the SOX6-reduced sensitivity to cisplatin could be turned over under the treatment of Baf A1, an autophagy-specific inhibitor. Further, the sensitivity to cisplatin could also be increased by inhibiting the expression of endogenous MAP4K4 under the treatment of PF-06260933, an MAP4K4-specific inhibitor. Taken together, the sensitivity of cervical cancer cells to cisplatin could be increased by inhibiting the SOX6-induced autophagy through both autophagy and MAP4K4 inhibitors. Since MAP4K4 was at the upstream of SOX6-induced autophagy, its inhibitor might be more specific in inhibiting autophagy and subsequently increasing the sensitivity of cervical cancer cells to cisplatin chemotherapy.

In conclusion, SOX6 could transactivate its direct target gene *MAP4K4* through binding to its specific binding sites located at the promoter of *MAP4K4* gene, then autophagy was induced by the MAP4K4-mediated activation of MAPK/ERK pathway and inhibition of PI3K-Akt-mTOR pathway. Further, the sensitivity to cisplatin treatment could be reduced by the SOX6-induced autophagy, and the endogenous SOX6 upregulated by cisplatin might contribute to the reduced sensitivity to cisplatin treatment. Moreover, the sensitivity of cervical cancer cells to cisplatin could be increased by inhibiting SOX6-induced autophagy through both autophagy and MAP4K4 inhibitors (Fig. 7C). This study uncovered the mechanism of SOX6-induced autophagy in cervical cancer cells and its role in regulation of the sensitivity to cisplatin chemotherapy, which might explain the underlying reason for the poor response of partial cervical cancer patients to the platinum-based chemotherapy. More importantly, this study shed new light on the usage of MAP4K4 inhibitors to increase the sensitivity of cervical cancer cells to the platinum-based chemotherapy.

## Materials and methods

### Cell lines

Human cervical cancer cell lines HeLa, CaSki and SiHa were used and were maintained in Dulbecco’s modified Eagle medium (DMEM) (Corning, Corning, New York, USA) supplemented with 10% fetal bovine serum (FBS) (PAN, Adenbach, Bavaria, Germany) in this study. HeLa-HA-SOX6-tet and HeLa-HA-SOX6ΔHMG-tet cell lines were constructed previously in our laboratory and were maintained in DMEM (Corning, Corning, New York, USA) supplemented with 10% FBS (PAN, Adenbach, Bavaria, Germany), 400 μg/mL G418 (Amresco, Solon, Washington, USA) and 4 μg/mL blasticidin (Thermo Fisher Scientific, Waltham, Massachusetts, USA). Final concentration of 4 μg/mL of doxycycline (Merck, Kenilworth, New Jersey, USA) was added into the culture medium of HeLa-SOX6-tet and HeLa-SOX6ΔHMG-tet cells to induce the expression of SOX6 and SOX6ΔHMG, respectively.

HeLa cells with endogenous *SOX6* gene knockout (HeLa-SOX6KO) were constructed in our laboratory. Specifically, HeLa cells were seeded into six-well plates and transduced with lentivirus that was produced by HEK 293FT cells transfected with lenti-CRISPR-SOX6 plasmids, and together with 10 μg/mL polybrene (Santa Cruz Biotechnology, Santa Cruz, CA, USA). After 48 h of infection, the infected HeLa cells were maintained in DMEM supplemented with 10% FBS and 2 μg/mL puromycin (Thermo Fisher Scientific, Waltham, Massachusetts, USA) for another 48 h, and then 100 cells were counted and subsequently seeded into a 96-well plate. Several single clones of HeLa cells were subsequently identified by sequencing of PCR product and western blotting to establish HeLa cells with endogenous *SOX6* gene knockout. All cell lines used in our research were tested negative for mycoplasma contamination.

### Cervical cancer tumor samples

Total 14 cervical cancer patients from the Gynecology Department of the Third Hospital of Peking University were included in our research, all of whom were diagnosed to be at local advanced stage and had received routine treatment by cisplatin combined with paclitaxel for 2 courses. The cervical cancer tissues from 14 cervical cancer patients were collected, half of whom were sensitive to chemotherapy according to the new guidelines to evaluate the response to treatment in solid tumors. This study was approved by the Ethics Committee of Third Hospital of Peking University. Written informed consent was obtained from each patient.

### Plasmids

The plex-HA-SOX6, plex-HA-SOX6ΔHMG, GFP-LC3, and PRL-TK plasmids were constructed previously and maintained in our laboratory. The pGL3-MAP4K4-promoter was constructed by inserting the promoter sequence of *MAP4K4* gene into pGL3-basic plasmid. Specifically, the promoter sequence of *MAP4K4* gene was amplified by PCR from HeLa DNA, and the PCR products were subsequently ligated into the pGL3-basic plasmid after digestion with *Kpn* I and *Hind* III. The pGL3-MAP4K4-promoter mutant 1 and pGL3-MAP4K4-promoter mutant 2 were constructed on the basis of pGL3-MAP4K4-promoter plasmid by the site directed rapid mutagenesis methods. The lenti-CRISPR-SOX6 plasmid was constructed by ligating the annealed oligonucleotides of *SOX6* gene-specific gRNA into the lenti-CRISPRv2 plasmid digested with *Bsm* BI. The pCDH-3×flag-MAP4K4 plasmid was constructed by ligating the synthesized sequence of the MAP4K4 CDS sequence containing 3×flag tag sequence into the pCDH-vector plasmid after digestion with *Bam* HI and *Xba* I. The primer sequences used for plasmid construction were listed in Supplementary Table S1.

### Antibodies and reagents

The following primary antibodies were purchased from Abcam: anti-SOX6 (ab30455), anti-p62/SQSTM1 (ab109012). The anti-HA-tag (M180-3) antibody was purchased from MBL International. The following antibodies were purchased from Cell Signaling Technology: anti-LC3B (2775 S), anti-p44/42 MAPK (9102 S), anti-phospho-p44/42 MAPK (4370 S), anti-Akt (2920 S), anti-phospho- Akt (4060 S), anti-mTOR (2983 S), anti-phospho-mTOR (5536 S), anti-p70 S6 kinase (2708 S), anti-phospho-p70 S6 kinase (9205 S), anti-PARP (9542), anti-caspase 9 (9502), anti-Bax (5023) and anti-Bcl-2 (15071). The following antibodies were purchased from Santa Cruz Biotechnology: anti-MAP4K4 (sc-100445) and anti-phospho-p38 (sc-166182). Second antibodies were IRDye 680LT donkey anti-rabbit IgG (LICOR, 926-68023), IRDye 800CW donkey anti-mouse IgG (LICOR, 926-32212), HRP-linked anti-rabbit IgG (Cell Signaling technology, 7074 S) and HRP-linked anti-mouse IgG (Cell Signaling technology, 7076 S). The antibodies mentioned above were all used for western blotting analyses. The following antibodies were used in immunofluorescence: anti-SOX6 (Abcam, ab30455), anti-p62/SQSTM1 (Cell Signaling technology, 88588 S), FITC-conjugated goat anti-mouse IgG (Zhongshan Golden Bridge, ZF-0312), FITC-conjugated goat anti-rabbit IgG (Zhongshan Golden Bridge, ZF-0311), TRITC-conjugated goat anti-mouse IgG (Zhongshan Golden Bridge, ZF-0313) and TRITC-conjugated goat anti-rabbit IgG (Zhongshan Golden Bridge, ZF-0316). The following antibodies were used in ChIP: anti-HA-tag (Abcam, ab9110) and IgG from rabbit serum (Sigma-Aldrich, I5006). Other regents used in this study were: bafilomycin A1 (Baf A1; Santa Cruz Biotechnology, sc-201550), PF-06260933 (TargetMol, T4221) and cisplatin (Pharmabiology, C21384).

### Microarray analysis

Total RNA was extracted by TRIzol^®^ Reagent (Thermo Fisher Scientific, Waltham, Massachusetts, USA) according to the manufacturer’s instructions. After identification of the purity and integrity, RNA samples were sent for microarray analysis carried out by Shanghai Biotechnology Corporation. Data were analyzed by the online SBC analysis system (SAS; http://sas.ebioservice.com). KEGG signaling pathway enrichment analysis and gene ontology (GO) enrichment analysis were carried out by the DAVID (http://david.abcc.ncifcrf.gov).

### RNA-sequencing analysis

Total RNA was extracted from the tissue using TRIzol^®^ Reagent (Thermo Fisher Scientific, Waltham, Massachusetts, USA) according to the manufacturer’s instructions, and the genomic DNA was removed using DNase I (TaKara, Akishima, Tokyo, Japan). Then RNA quality was determined by 2100 Bioanalyser (Agilent) and quantified using the ND-2000 (Thermo Fisher Scientific, Waltham, Massachusetts, USA). Only high-quality RNA sample (OD260/280 = 1.8–2.2, OD260/230 ≥ 2.0, RIN ≥ 6.5, 28 S:18 S ≥ 1.0, >2 μg) was used to construct sequencing library and sequencing carried out by Majorbio corporation. Functional enrichment analysis including KEGG and GO were analyzed on the free online platform of Majorbio Cloud Platform (www.majorbio.com). The raw RNA-sequencing data have been deposited in the Genome Sequence Archive in National Genomics Data Center, China National Center for Bioinformation under the accession number of HRA001566.

### RNA interference

HeLa-SOX6-tet cells were transfected with MAP4K4-specific siRNA oligonucleotide duplex (RiBobio, Guangzhou, Guangdong, China) using Lipofectamine RNAiMAX (Thermo Fisher Scientific, Waltham, Massachusetts, USA). Briefly, siRNA and Lipofectamine RNAiMAX were added into Opti-MEM medium (Thermo Fisher Scientific, Waltham, Massachusetts, USA) in turn, then mixed them gently and incubated for 20 min at room temperature. Next, the mixture was added to the culture medium at a final concentration of 50 nM siRNA. The effectiveness of siRNA knockdown was evaluated by reverse-transcription and quantitative polymerase chain reaction (RT-qPCR) and western blotting at 3 days post transfection.

### RNA extraction, reverse transcription, and qPCR

Total RNA was extracted using TRIzol^®^ Reagent (Thermo Fisher Scientific, Waltham, Massachusetts, USA) according to the manufacturer’s instructions. Reverse transcription was performed using Transcriptor First Strand cDNA Synthesis Kit (Roche, Basel, Kanton Basel, Switzerland). The primer used for reverse transcription was random primer. The mRNA levels of MAP4K4, SOX6, and ACTB were detected by qPCR (SYBR Green method) using an Applied Biosystems StepOne plus Real-Time PCR system (Thermo Fisher Scientific, Waltham, Massachusetts, USA). The primers used to detect the mRNA levels of MAP4K4, SOX6 and ACTB were listed in Supplementary Table S2.

### Chromatin immunoprecipitation assay (ChIP)

The ChIP assay was performed using Simple ChIP Enzymatic chromatin IP kit (Cell Signaling Technology, Danvers, Massachusetts, USA) as previously described [50]. Briefly, HeLa cells were transfected with plex-HA-SOX6 or plex-HA-SOX6ΔHMG plasmid. At 48 h post transfection, cells were treated with 1% methanol at room temperature for 10 min, and then added glycine to stop the reaction. After washing with cold PBS for three times, cells were lysed with SDS lysis buffer, and then the lysates were fragmented by ultrasound to get 300–500 bp DNA fragments in length. Finally, the lysates were incubated with rabbit anti-HA-tag-ChIP grade (Abcam, ab9110) or IgG from rabbit serum (Sigma-Aldrich, I5006).

### Dual-luciferase reporter assay

At 48 h post transfection, cells were washed in PBS and lysed with passive lysate buffer (Promega, Madison, Wisconsin, USA), and then the relative luciferase activity was measured using dual-luciferase assay kit (Promega, Madison, Wisconsin, USA) following the manufacturer’s instructions.

### Xenograft implantation in vivo

HeLa-HA-SOX6-tet and HeLa-HA-SOX6ΔHMG-tet cells were subcutaneously injected into the left flank of 12 BALB/c female nude mice (3 × 10^6^ cells for each mouse), respectively. Subsequently, these mice were randomly divided into two groups, one group of them (12 mice) were daily intraperitoneally injected with Dox (20 mg/kg), and the other group (12 mice) were daily intraperitoneally injected with solvent control-phosphate buffered solution (PBS) for 3 weeks, and the tumor blocks were collected when the mice were sacrificed under anesthesia at 3 weeks post-injection.

For the investigation of influence of the SOX6-induced autophagy on the sensitivity to cervical cancer cells to cisplatin treatment, HeLa-HA-SOX6-tet cells were subcutaneously injected into the left flank of 32 BALB/c female nude mice (3 × 10^6^ cells for each mouse), then the mice were randomly divided into four groups (8 mice for each group) at 1-week post-injection and were daily intraperitoneally injected with Dox (20 mg/kg, PBS as solvent control) every day and cisplatin (3 mg/kg, saline as solvent control) every other day for the next 2 weeks. The sizes of tumors in mice were measured every week, and the tumor blocks were collected when the mice were sacrificed under anesthesia at 3 weeks post-injection. This study was approved by the Ethics Committee of Peking University Health Science Center.

### Western blotting

Cells were harvested and lysed in RIPA lysis buffer, and the lysate was subsequently subjected to polyacrylamide gel electrophoresis. The proteins in polyacrylamide gel were then transferred onto a PVDF membrane by the method of wet electrophoretic transfer. The membrane was then blocked in 5% skimmed milk for 1 h and incubated with the primary antibodies at 4 °C overnight. After washing in TBST for 3 times, the membrane was incubated with the secondary antibodies for 1 h at room temperature. Then the levels of proteins were detected by Odessey infrared imaging system (LICOR, Lincoln, Nebraska, USA) or chemiluminescence imaging analysis system (Tanon, Shanghai, China).

### Immunofluorescence staining

Cells were washed with cold PBS for 3 times and were fixed with 4% paraformaldehyde at room temperature for 20 min, followed by permeabilized with 0.5% Triton X-100 at room temperature for 15 min. Then cells were blocked in 20% goat serum at room temperature for 1 h and incubated with the primary antibody at 4 °C overnight. After washed with PBS for 3 times, cells were then incubated with the secondary antibodies at room temperature for 1 h, followed by staining with Hoechst 33342. At last, cells were examined under a TCS-SP8 STED confocal laser scanning microscope (Leica, Frankfurt, Hesse-Darmstadt, Germany) or an inverted fluorescent microscope (Leica, Frankfurt, Hesse-Darmstadt, Germany).

The immunofluorescent staining was double-blinded and carried out by Wuhan Servicebio technology cooperation (Wuhan, Hubei, China). The tissue samples of the xenograft and the cervical cancer patients were solidified in liquid nitrogen, and were sent for the immunofluorescent staining. The areal density (IOD/Area) was calculated by image-pro plus 6.0 software (Media Cybernetics, Inc., Rockville, MD, USA) to reflect the relative protein level.

### Apoptosis assay

Apoptotic cells were detected using Annexin V-FITC/PI apoptosis detection kit (Dojindo, Kumamoto, Kyushu, Japan) according to the manufacturer’s instructions. The early apoptotic cells were stained with Annexin V-FITC, and the late apoptotic cells were stained with propidium iodide (PI). The percentage of apoptotic cells were analyzed and calculated with Flowjo software (v7.6, Stanford, California, USA). The cell apoptosis in xenograft was detected by Terminal deoxynucleotidyl transferase (TdT) dUTP Nick-End Labeling (TUNEL) assay on frozen sections, which were carried out by Wuhan Servicebio technology cooperation.

### Transmission electron microscopy

Cells were harvested and fixed with 2.5% glutaraldehyde followed by stained with 1% osmic acid. Next, cells were dehydrated with different concentrations of acetone, and then were embed and stained with lead citrate and uranyl acetate. Autophagic structures were then detected using a JEM-1400 transmission electron microscope (JEOL, Akishima, Tokyo, Japan).

### Statistical analysis

All data were presented as mean±standard error of mean (SEM). Statistical analysis was performed using GraphPad prism 8.0 software (GraphPad Software, San Diego, California, USA). Comparisons among groups were analyzed by one-way ANOVA and post hoc Tukey tests, and the differences between groups were analyzed by two-tailed Student’s *t*-tests, and a *P*-value less than 0.05 was considered statistically significant.

## Supplementary information

Supplementary materials for MAP4K4 mediates the SOX6-induced autophagy and reduces the chemosensitivity of cervical cancer.

Reproducibility Checklist

## Data Availability

All data needed to evaluate the conclusions in the paper are present in the paper and/or the Supplementary Materials. Additional data related to this paper are available on reasonable request.
