# Supplementary material for: MAP4K4 mediates the SOX6-induced autophagy and reduces the chemosensitivity of cervical cancer
Source: Cell Death Dis. 2021 Dec 20;13(1):13. doi: 10.1038/s41419-021-04474-1 (PMC8688448; doi:10.1038/s41419-021-04474-1)
Supplement: Supplementary file 1 — Supplementary materials for MAP4K4 mediates the SOX6-induced autophagy and reduces the chemosensitivity of cervical cancer. [file 41419_2021_4474_MOESM1_ESM.docx]

**MAP4K4 mediates the SOX6-induced autophagy and reduces the chemosensitivity of cervical cancer.**

Hongxin Huang^1*^, Qin Han^2*^, Han Zheng^1^, Mingchen Liu^1^, Shu Shi^1^, Ting Zhang^1^, Xingwen Yang^1^, Zhongqing Li^1^, Qiang Xu^1^, Hongyan Guo^2#^, Fengmin Lu^1^, Jie Wang^1#^

^*^These authors contributed equally to this work.

^#^Corresponding Authors:

Jie Wang, MD & Associate Professor

Department of Microbiology & Infectious Disease Center, School of Basic Medical Sciences, Peking University Health Science Center, 38 Xueyuan Road, Haidian District, Beijing 100191, P.R. China; E-mail: wangjie2015@hsc.pku.edu.cn; Tel: 86-10-82805137.

Hongyan Guo, MD & Professor

Department of Gynecology and Obstetrics, The Third Hospital of Peking University, Beijing 100191, P.R. China; E-mail: bysyghy@163.com; Tel: 86-10-82267510.

**
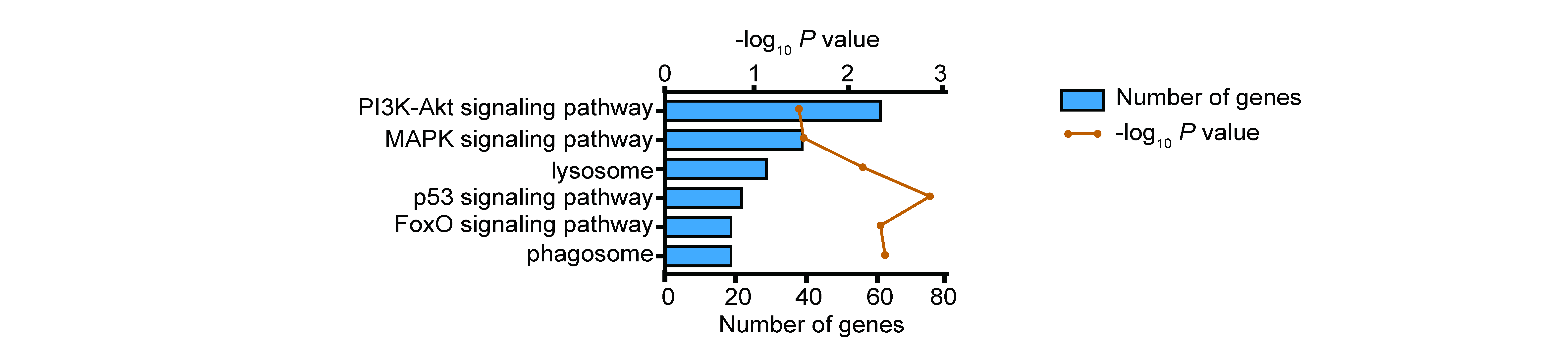
**

**Figure S1. Six** **pathways related to the SOX6-induced autophagy were screened.** According to the differentially expressed genes (DEGs) of microarray data, six of twenty enriched KEGG pathways regulated by SOX6 were associated with autophagy machinery.

**
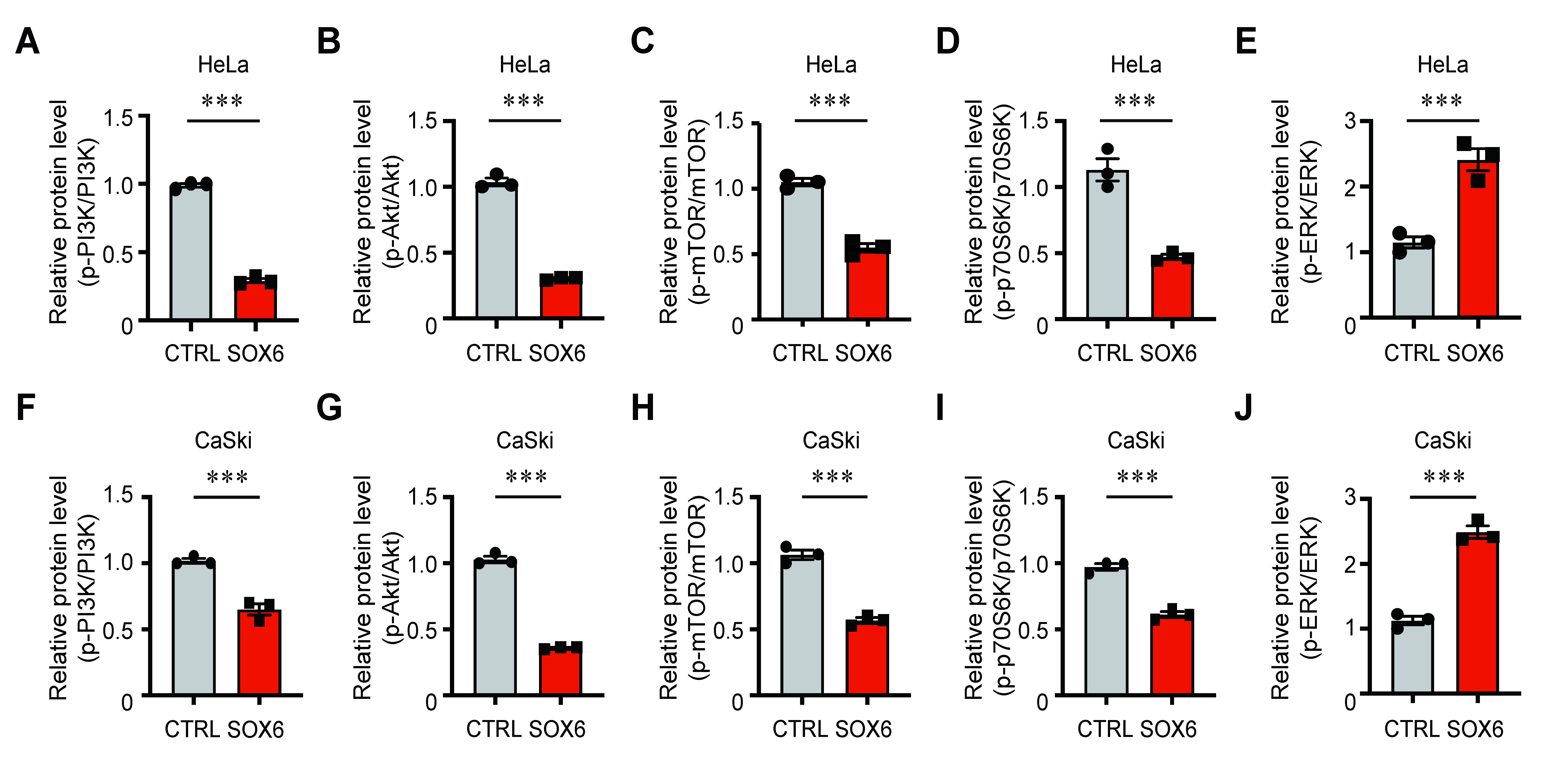
**

**Figure S2. Quantification of relative proteins levels presented in Fig. 2B by gray value analyses.** (**A**) Quantification on the relative levels of the p-PI3K, (**B**) p-Akt, (**C**) p-mTOR, (**D**) p-p70S6K and (**E**) p-ERK in HeLa cells transfected with plex-HA-SOX6 expression plasmid or empty vector control (CTRL). (**F**) Quantification on the relative levels of the p-PI3K, (**G**) p-Akt, (**H**) p-mTOR, (**I**) p-p70S6K and (**J**) p-ERK in CaSki cells transfected with plex-HA-SOX6 expression plasmid or vector control (CTRL). Data are mean±SEM of three independent experiments. (****P*<0.001, Student’s *t*-test, two tails).


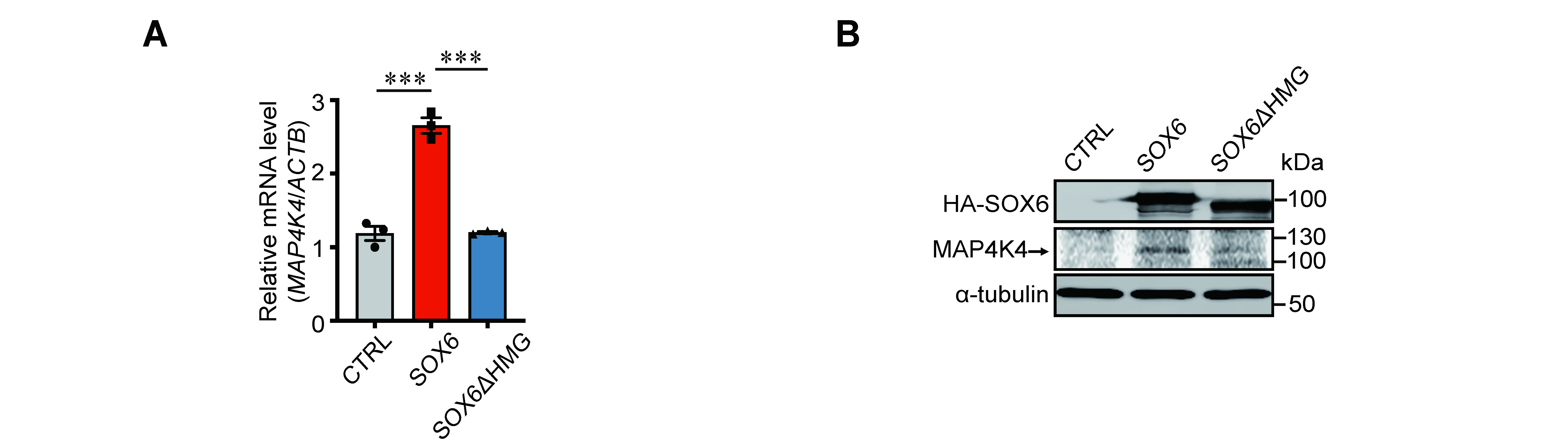


**Figure S3. The mRNA and protein levels of MAP4K4 were up-regulated by SOX6 in HeLa cells.** (**A**) The RT-qPCR (SYBR Green) analyses on the levels of MAP4K4 mRNA in HeLa cells transfected with plex-MCS (CTRL), plex-HA-SOX6 or plex-HA-SOX6ΔHMG expression plasmid, respectively. β-Actin (ACTB) mRNA was used as the internal control. (**B**) Western blotting analyses on the levels of MAP4K4 protein in HeLa cells transfected with plex-MCS (CTRL), plex-HA-SOX6 or plex-HA-SOX6ΔHMG expression plasmid, respectively. α-tubulin protein was used as the internal control. Data are mean±SEM of three independent experiments. (****P*<0.001, one-way ANOVA and post hoc Tukey tests).

**
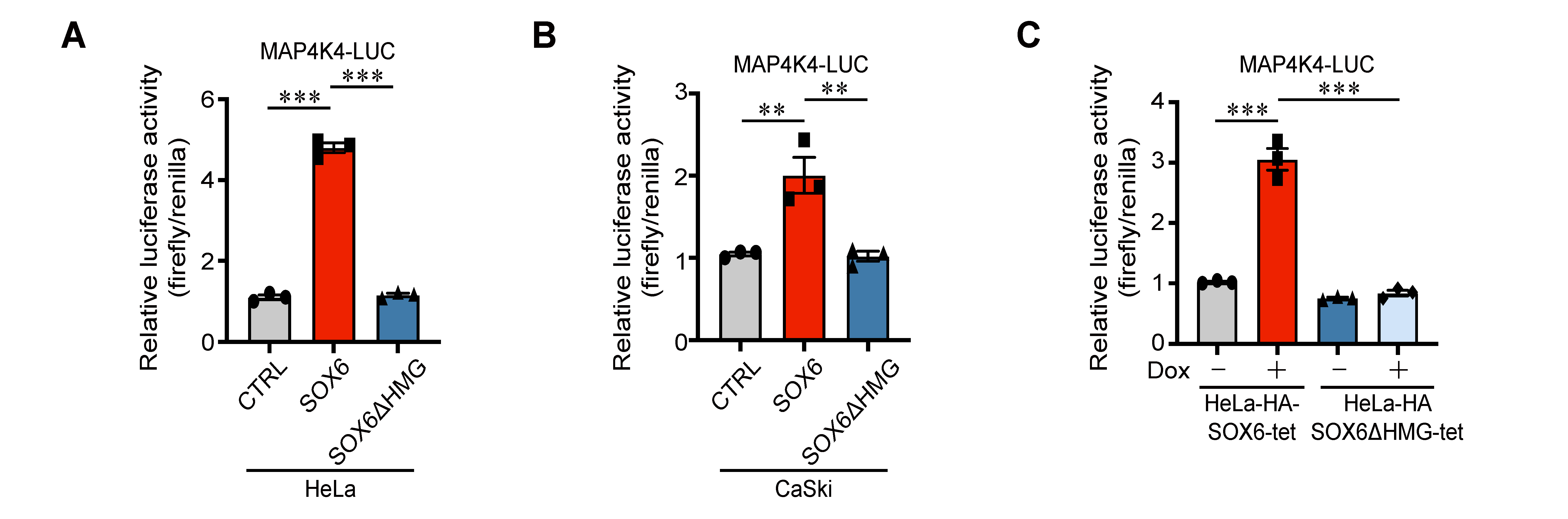
**

**Figure S4. SOX6 enhances the transcriptional activity of *MAP4K4* gene promoter.** (**A**) Dual-luciferase assays on the effects of SOX6 in the transcriptional activity of *MAP4K4* gene promoter in HeLa and **(B**) CaSki cells co-transfected with pGL3-MAP4K4-promoter, PRL-TK and plex-MCS (CTRL), plex-HA-SOX6 or plex-HA-SOX6ΔHMG expression plasmids. (**C**) Dual-luciferase assay on the effects of SOX6 in the transcriptional activity of *MAP4K4* gene promoter in HeLa-HA-SOX6-tet and HeLa-HA-SOX6ΔHMG-tet cells co-transfected with pGL3-MAP4K4-promoter and PRL-TK plasmids and subsequently treated with or without Dox (4 μg/mL). Data are mean±SEM of three independent experiments. (***P*<0.01, ****P*<0.001, one-way ANOVA and post hoc Tukey tests).

**
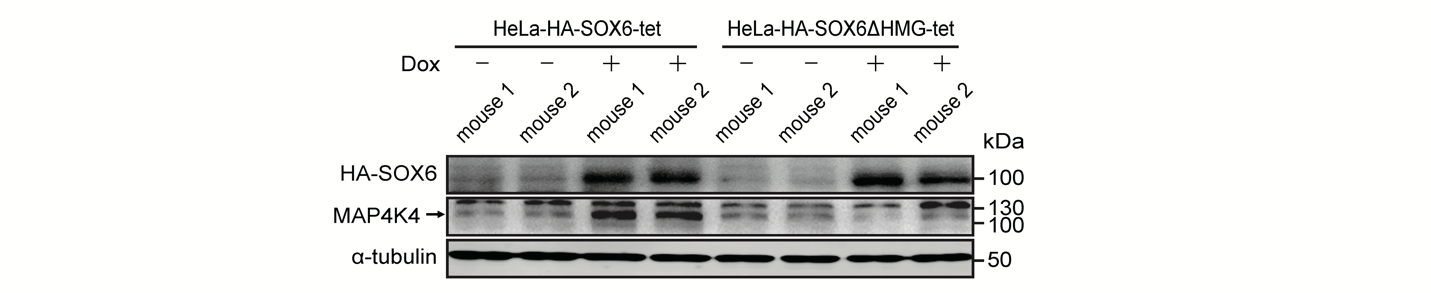
**

**Figure S5. SOX6 upregulates MAP4K4 expression in xenograft depending on its HMG domain.** Western blotting analysis on the protein levels of SOX6 and MAP4K4 in xenograft produced by subcutaneous injection of HeLa-HA-SOX6-tet or HeLa-HA-SOX6ΔHMG-tet cells into the left flank of nude mice, after which the mice were daily intraperitoneally injected with Dox (20 mg/kg, PBS as solvent control) for three weeks. α-tubulin protein was used as the internal control.


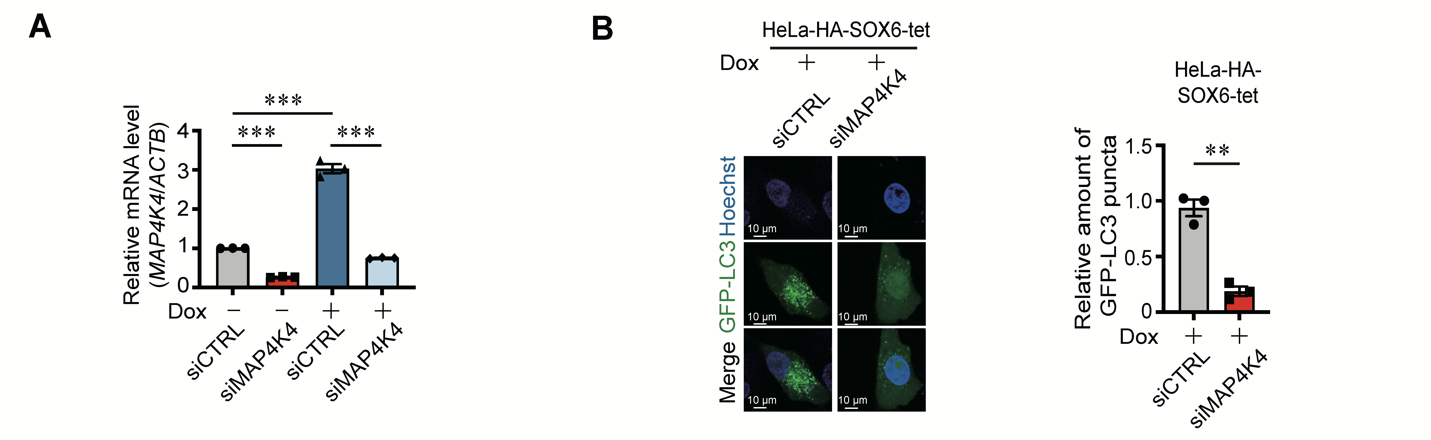


**Figure S6. siRNA targeting MAP4K4 inhibits the SOX6-induced autophagosome formation in HeLa-HA-SOX6-tet cells.** (**A**) The RT-qPCR (SYBR Green) analysis on the level of MAP4K4 mRNA in HeLa-HA-SOX6-tet cells transfected with siRNA targeting endogenous *MAP4K4* gene (siMAP4K4) or control siRNA (siCTRL) and subsequently treated with or without Dox (4 μg/mL). β-Actin (ACTB) mRNA was used as the internal control. Data are mean±SEM of three independent experiments (****P*<0.001, one-way ANOVA and post hoc Tukey tests). (**B**) Representative confocal microscopy images of HeLa-HA-SOX6-tet cells co-transfected with GFP-LC3 and siRNA targeting endogenous *MAP4K4* gene (siMAP4K4) or control siRNA (siCTRL) and subsequently treated with Dox (4 μg/mL). Data are mean±SEM of three independent experiments and at least 50 cells scored (***P*<0.01, Student’s *t*-test, two tails).

**

**

**Figure S7. Quantification of relative proteins levels presented in Fig. 4A and 4B by gray value analyses.** (**A**) Quantification on the relative levels of the p-PI3K, (**B**) p-Akt, (**C**) p-mTOR, (**D**) p-p70S6K and (**E**) p-ERK in HeLa-HA-SOX6-tet and HeLa-HA-SOX6ΔHMG-tet cells treated with or without Dox (4 μg/mL). Data are mean±SEM of three independent experiments (***P*<0.01, ****P*<0.001, one-way ANOVA and post hoc Tukey tests). (**F**) Quantification on the relative levels of the p-PI3K, (**G**) p-Akt, (**H**) p-mTOR, (**I**) p-p70S6K and (**J**) p-ERK in HeLa-HA-SOX6-tet cells transfected with siRNA targeting endogenous *MAP4K4* gene (siMAP4K4) or control siRNA (siCTRL) and subsequently treated with Dox (4 μg/mL). Data are mean±SEM of three independent experiments (***P*<0.01, ****P*<0.001, Student’s *t*-test, two tails).

**
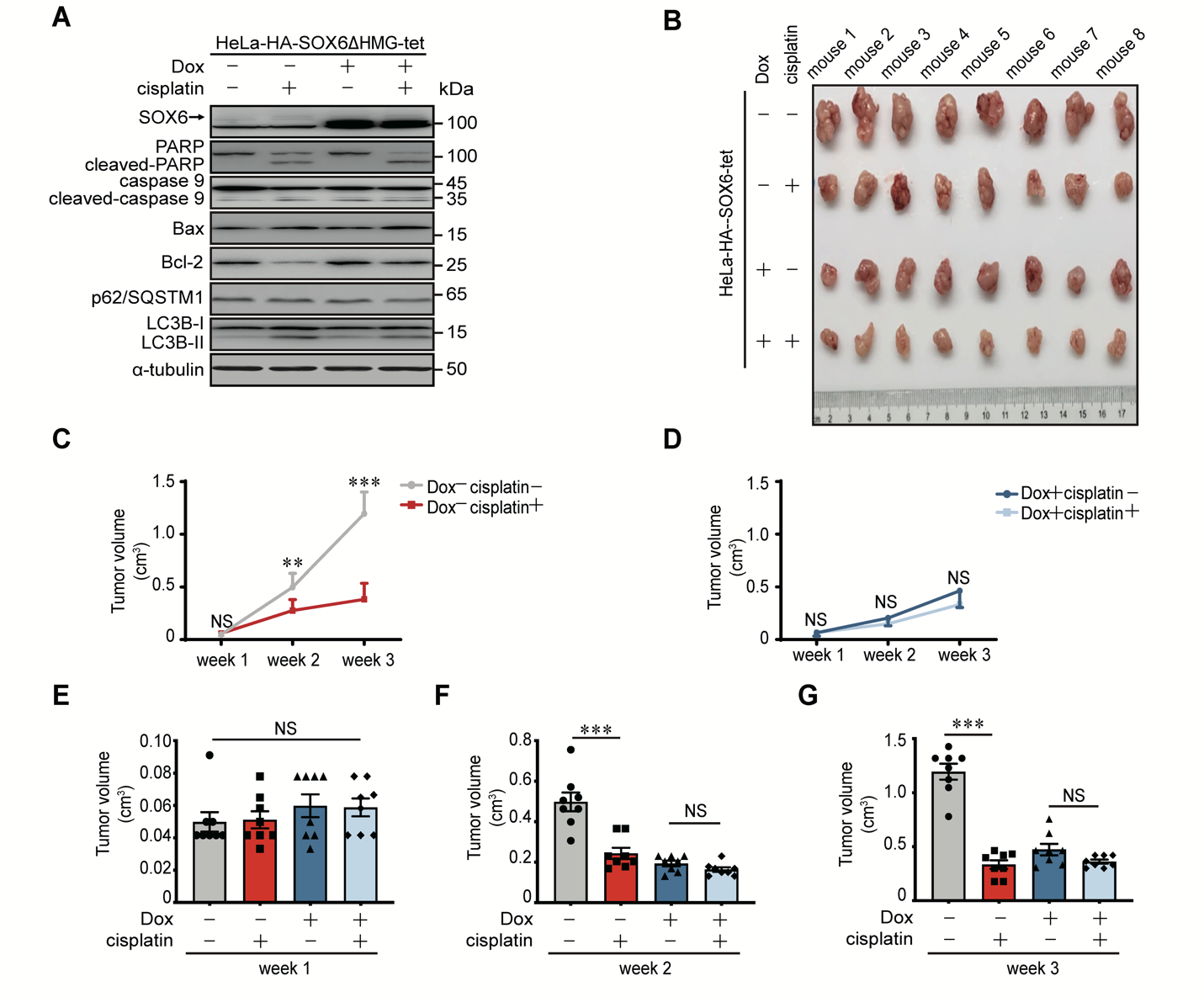
**

**Figure S8. SOX6-induced autophagy reduces the sensitivity of cervical cancer cells to cisplatin treatment *in vitro* and *in vivo*.** (**A**) Western blotting analysis on the protein levels of PARP, caspase 9, Bax, Bcl-2, p62/SQSTM1 and LC3B in HeLa-HA-SOX6ΔHMG-tet cells with or without cisplatin (20 μM) treatment. α-tubulin protein was used as the internal control. (**B**) Representative tumor blocks that were collected from the mice sacrificed under anesthesia at 3 weeks post-injection. Growth curve of tumors formed by subcutaneous injection of HeLa-HA-SOX6-tet cells into the left flank of nude mice, one week after which the mice were daily intraperitoneally injected with (**C**) Dox (20 mg/kg) and (**D**) PBS (solvent control) together with injection of cisplatin every other day (3 mg/kg, saline as solvent control) for the next two weeks. Average tumor weights (g) of tumor blocks measured at week 1 (**E**), week 2 (**F**) and week 3 (**G**). Data are the mean±SEM. (***P*<0.01, ****P*<0.001, NS, non-significant, Student’s *t*-test, two tails for **C** and **D**, one-way ANOVA and post hoc Tukey tests for **E**, **F** and **G**).

**
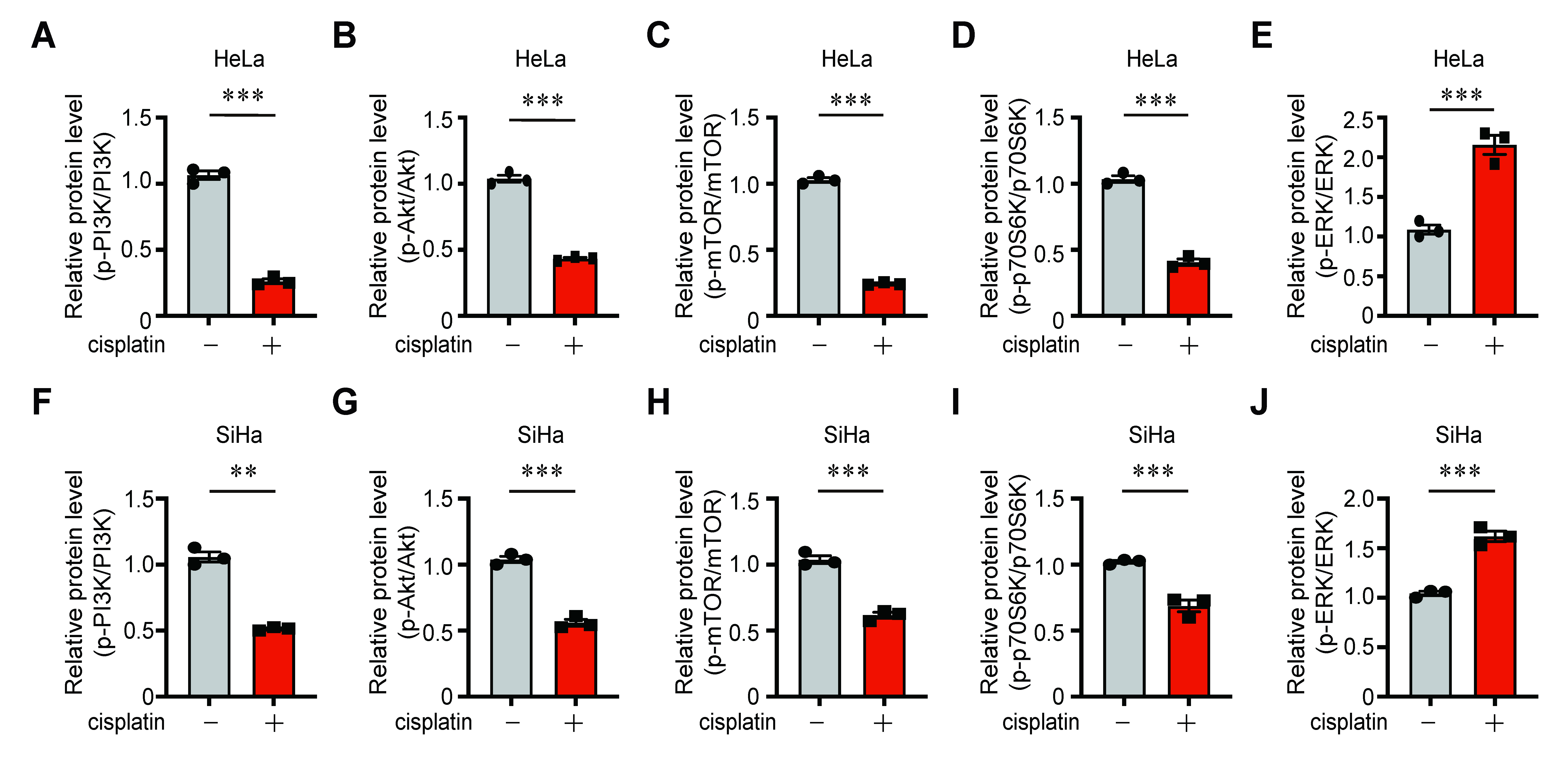
**

**Figure S9. Quantifications of relative proteins levels presented in Fig. 6B by gray value analyses.** (**A**) Quantification on the relative levels of the p-PI3K, (**B**) p-Akt, (**C**) p-mTOR, (**D**) p-p70S6K and (**E**) p-ERK in HeLa cells treated with or without cisplatin (20 μM). (**F**) Quantification on the relative levels of the p-PI3K, (**G**) p-Akt, (**H**) p-mTOR, (**I**) p-p70S6K and (**J**) p-ERK in SiHa cells treated with or without cisplatin (20 μM). Data are mean±SEM of three independent experiments. (***P*<0.01, ****P*<0.001, Student’s *t*-test, two tails).

**
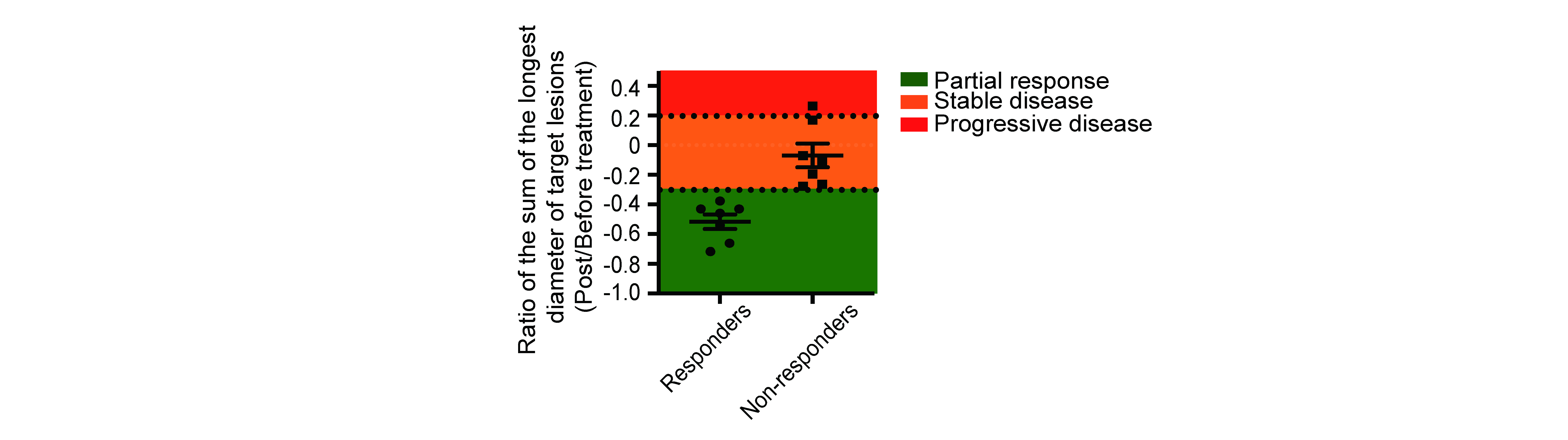
**

**Figure S10. Clinical characteristics of the** **cervical cancer tissues collected from 14 cervical cancer patients.** The cervical cancer tissues collected from 14 cervical cancer patients after receiving routine cisplatin treatment were divided into partial response (indicated by the decreased percentage on the sum of the longest diameter of target lesions more than 30%), stable disease (indicated by the changed percentage on the sum of the longest diameter of target lesions from decreased 30% to increased 20%) and progressive disease (indicated by the increased percentage on the sum of the longest diameter of target lesions more than 20%).

**Table S1. The sequences of primers used in vector construction.**

| Primer names | Primer sequences（5′-3′） |
| --- | --- |
| pGL3-MAP4K4-promoter | F: CGGGGTACCCACTCCACTGCACTCCAGCTTG |
|  | R: CCCAAGCTTGGGGCCCTTAGTACCAACCC |
| pGL3-MAP4K4-mutant 1F | CTAACTACCGACTGGCTTCCGAGTATGCCGGAAGAAACCTGTTGGTTGA |
| pGL3-MAP4K4-mutant 1R | TCAACCAACAGGTTTCTTCCGGCATACTCGGAAGCCAGTCGGTAGTTAG |
| pGL3-MAP4K4-mutant 2F | TGGCTTCCGAGTATGCCGGAAGATGTCTGTTGGTTGATCCTTAGTGATC |
| pGL3-MAP4K4-mutant 2R | GATCACTAAGGATCAACCAACAGACATCTTCCGGCATACTCGGAAGCCA |
| pGL3-MAP4K4-mutant identify 1F | ACTACCGACTGGCTTCCGAGTAT |
| pGL3-MAP4K4-mutant identify 2F | CTTCCGAGTATGCCGGAAGATGT |
| pGL3-MAP4K4-mutant identify R | CTTCATAGCCTTATGCAGTTCGT |
| lenti-CRISPR-SOX6 gRNA 1F | CACCGCAAATGGAGAGGTGGCTTGCT |
| lenti-CRISPR-SOX6 gRNA 1R | AAACAGCAAGCCACCTCTCCATTTG |
| lenti-CRISPR-SOX6 gRNA 2F | CACCGACCATTCAACAAGATGCTGAC |
| lenti-CRISPR-SOX6 gRNA 2R | AAACGTCAGCATCTTGTTGAATGGT |
| lenti-CRISPR-SOX6 gRNA identify F | AGCAAAGAGGGAAAGAAGGACA |
|  |  |
|  |  |
| Primer names | Primer sequences（5′-3′） |
| lenti-CRISPR-SOX6 gRNA identify R | CCATTTTTCAAGTTTTTCCAGTTAT |
| pCDH-3×flag-MAP4K4 F | CGCGGATCCGCCGCCACCATGGACTACAAAGACCATGACGGTGATTATAAAGATCATGACATCGACTACAAGGATGACGATGACAAGATGGCGAACGACTCCCCT |
| pCDH-3×flag-MAP4K4 R | GCTCTAGACTACCAGCTCAGAAGAGAAGTCCT |

F: forward, R: reverse.

**Table S2. The primer sequences used in quantitative PCR.**

| Gene name | Primer sequences（5′-3′） |
| --- | --- |
| *MAP4K4* | F: TCTGGCTTGTTATGGAGTTCTGTG |
|  | R: TGTTCTTCACAAGGTCTGTAATGGA |
| *ACTB* | F: CTACAGCTTCACCACCACGG |
|  | R: TCAGGCAGCTCGTAGCTCTTC |
| *SOX6* | F: GGACAGCGTTCTGTCATCTC |
|  | R: CTCTTGTTCAGTCCGAGTCA |

F: forward, R: reverse.
