# Supplementary material for: MAP4K4 mediates the SOX6-induced autophagy and reduces the chemosensitivity of cervical cancer
Source: Cell Death Dis. 2021 Dec 20;13(1):13. doi: 10.1038/s41419-021-04474-1 (PMC8688448; doi:10.1038/s41419-021-04474-1)
Supplement: Supplementary file 2 — Reproducibility Checklist [file 41419_2021_4474_MOESM2_ESM.pdf]

Corresponding Author Name: Jie Wang
Manuscript Number: CDDIS-21-379[illegible]

SPRINGER NATURE

Reporting Summary

Springer Nature wishes to improve the reproducibility of the work that we publish. This checklist is used to ensure good reporting standards and to improve the reproducibility. Please respond completely to all questions relevant to your manuscript. For more information, please read the journal's Guide to Authors.

☑ **Check here to confirm that the following information is available in the Material & Methods section:**

- **The exact sample size (*n*)** for each experimental group/condition, given as a number, not a range
- **A description of the sample collection** allowing the reader to understand whether the samples represent technical or biological replicates (including how many animals, litters, culture, etc.)
- **A statement of how many times the experiment shown was replicated in the laboratory**
- **Definitions of statistical methods and measures**: For small sample sizes (n<5) descriptive statistics are not appropriate, instead plot individual data points
  - Very common tests, such as *t*-test, simple χ^2 tests, Wilcoxon and Mann-Whitney tests, can be unambiguously identified by name only, but more complex techniques should be described in the methods section
  - Are tests one-sided or two-sided?
  - Are there adjustments for multiple comparisons?
  - **Statistical test results**, e.g., ***P* values**
  - Definition of **'center values'** as **median or mean**;
  - Definition of **error bars as s.d. or s.e.m. or c.i.**

Please ensure that the answers to the following questions are reported in the manuscript itself. We encourage you to include a specific subsection in the methods section for statistics, reagents and animal models. Below, provide the page number or section and paragraph number.

Statistics and general methods

	Reported in section/paragraph or page #
1. How was the sample size chosen to ensure adequate power to detect a pre-specified effect size? (Give section/paragraph or page #)	The sample size was estimated from preliminary experiments or from reports in the literature.
For animal studies, include a statement about sample size estimate even if no statistical methods were used.	The sample size was estimated from preliminary experiments or from reports in the literature.
2. Describe inclusion/exclusion criteria if samples or animals were excluded from the analysis. Were the criteria pre-established? (Give section/paragraph or page #)	No data was excluded.
3. If a method of randomization was used to determine how samples/animals were allocated to experimental groups and processed, describe it. (Give section/paragraph or page #)	All the nude mice used in this study were chosen randomly (Page Number 23, Line 491 and 500). And after the subcutaneously injection of the corresponding cells into the left flank of nude mice, the mice were randomly [illegible]
For animal studies, include a statement about randomization even if no randomization was used.	Page Number 23, Line 491 and 500.

4. If the investigator was blinded to the group allocation during the experiment and/or when assessing the outcome, state the extent of blinding. (Give section/paragraph or page #)	No blinding was performed for data collection.
For animal studies, include a statement about blinding even if no blinding was done.	No blinding was performed for data collection.
5. For every figure, are statistical tests justified as appropriate?	Yes, page number 35 to 41.
Do the data meet the assumptions of the tests (e.g., normal distribution)?	Yes, page number 35 to 41.
Is there an estimate of variation within each group of data?	Yes, the variation has been marked in each figure.
Is the variance similar between the groups that are being statistically compared? (Give section/paragraph or page #)	Yes, the variance is similar between the groups that are being statistically compared, page number 34 to 40.

Reagents

	**Reported in section/paragraph or page #**
6. Report the source of antibodies (vendor and catalog number)	Materials and methods section, page number 19-20.
7. Identify the source of cell lines and report if they were recently authenticated (e.g., by STR profiling) and tested for mycoplasma contamination	All the cell lines were authenticated previously and tested for mycoplasma contamination, page number 18.

Animal Models

	**Reported in section/paragraph or page #**
8. Report species, strain, sex and age of animals	Materials and methods section, page number 23.
9. For experiments involving live vertebrates, include a statement of compliance with ethical regulations and identify the committee(s) approving the experiments.	All xenograft experiments were approved by the Ethics Committee of Peking University Health Science Center (Page 23).

10. We recommend consulting the ARRIVE guidelines (*PLoS Biol.* **8**(6), e1000412,2010) to ensure that other relevant aspects of animal studies are adequately reported.

Human subjects

	Reported in section/paragraph or page #
11. Identify the committee(s) approving the study protocol.	This study was approved by the Ethics Committee of Third Hospital of Peking University (Page 18, Line 388-389).
12. Include a statement confirming that informed consent was obtained from all subjects.	Written informed consent was obtained from each patient (Page 18, Line 388-389).
13. For publication of patient photos, include a statement confirming that consent to publish was obtained.	Not applicable.
14. Report the clinical trial registration number (at ClinicalTrials.gov or equivalent).	Not applicable.

15. For phase II and III randomized controlled trials, please refer to the CONSORT statement and submit the CONSORT checklist with your submission.

16. For tumor marker prognostic studies, we recommend that you follow the REMARK reporting guidelines.

Data deposition

	Reported in section/paragraph or page #
17. Provide accession codes for deposited data. Data deposition in a public repository is mandatory for:<br>a. Protein, DNA and RNA sequences<br>b. Macromolecular structures<br>c. Crystallographic data for small molecules<br>d. Microarray data	The raw RNA-sequencing data have been deposited in the Genome Sequence Archive in National Genomics Data Center, China National Center for Bioinformation. The accession number is HRA001566 (Page 21, Line 449-451).

Deposition is strongly recommended for many other datasets for which structured public repositories exist; more details on our data policy are available in the Guide to Authors. We encourage the provision of other source data in supplementary information or in unstructured repositories such as Figshare and Dryad. We encourage publication of Data Descriptors (see Scientific Data) to maximize data reuse.

18. If computer code was used to generate results that are central to the paper's conclusions, include a statement in the Methods section under "**Code availability**" to indicate whether and how the code can be accessed. Include version information as necessary and any restrictions on availability.	Not applicable.
